# Characterization of novel small molecule inhibitors of estrogen receptor-activation function 2 (ER-AF2)

**DOI:** 10.1186/s13058-024-01926-2

**Published:** 2024-11-26

**Authors:** Jane Foo, Francesco Gentile, Shabnam Massah, Helene Morin, Kriti Singh, Joseph Lee, Jason Smith, Fuqiang Ban, Eric LeBlanc, Robert Young, Natalie Strynadka, Nada Lallous, Artem Cherkasov

**Affiliations:** 1 Vancouver Prostate Centre, Department of Urologic Science, University of British Columbia, 2660 Oak Street, Vancouver, BC V6H 3Z6 Canada; 2 Department of Chemistry, Simon Fraser University, Burnaby, BC V5A 1S6 Canada; 3 Department of Biochemistry and Molecular Biology and Centre for Blood Research, University of British Columbia, Vancouver, BC V6T 1Z3 Canada

**Keywords:** Estrogen receptor, Activation function 2, Small molecule inhibitors, Breast cancer

## Abstract

Up to 40% of patients with estrogen receptor (ER)-positive breast cancer will develop resistance against the majority of current ER-directed therapies. Resistance can arise through various mechanisms such as increased expression levels of coregulators, and key mutations acquired in the receptor’s ligand binding domain rendering it constitutively active. To overcome these resistance mechanisms, we explored targeting the ER Activation Function 2 (AF2) site, which is essential for coactivator binding and activation. Using artificial intelligence and the deep docking methodology, we virtually screened > 1 billion small molecules and identified 290 potential AF2 binders that were then characterized and validated through an iterative screening pipeline of cell-based and cell-free assays. We ranked the compounds based on their ability to reduce the transcriptional activity of the estrogen receptor and the viability of ER-positive breast cancer cells. We identified a lead compound, VPC-260724, which inhibits ER activity at low micromolar range. We confirmed its direct binding to the ER-AF2 site through a PGC1α peptide displacement experiment. Using proximity ligation assays, we showed that VPC-260724 disrupts the interaction between ER-AF2 and the coactivator SRC-3 and reduces the expression of ER target genes in various breast cancer models including the tamoxifen resistant cell line TamR3. In conclusion, we developed a novel ER-AF2 binder, VPC-260724, which shows antiproliferative activity in ER-positive breast cancer models. The use of an ER-AF2 inhibitor in combination with current treatments may provide a novel complementary therapeutic approach to target treatment resistance in ER-positive breast cancer.

**Supplementary Information:**

The online version contains supplementary material available at 10.1186/s13058-024-01926-2.

## Background

Breast cancer is the most prevalent malignancy in women, accounting for more than 2 million annual diagnoses globally [1]. Breast cancer is typically divided into four molecular subtypes: Luminal A, Luminal B, human epidermal growth factor receptor 2 (HER2)-positive, and triple negative [2]. Approximately 80% of cases fall within the luminal A or B categories, both characterized by the expression of the estrogen receptor (ER) [3], which drives their growth and response to hormone therapy. The HER2-positive subtype can be divided into two subgroups: HER2-enriched, which is negative for both ER and the progesterone receptor (PR), but positive for HER2, and the luminal B subtype, which can also have HER2 overexpression [4]. In contrast, the triple negative subtype lacks the expression of ER, PR, and HER2, making it more challenging to treat due to the absence of targeted receptors.

Two ER isoforms exist, ER⍺ and ERβ, with both full-length proteins organized into six highly conserved domains (A-F). These include the N-terminal domain (NTD) (A-B domains), which contains the Activation-Function 1 (AF1) —a key element for ligand-independent transcriptional activation [5]. The protein also includes the DNA-binding domain (DBD) in the C domain, the hinge region in the D domain, and the Ligand Binding Domain (LBD) in the E and F domains (Supplementary Figure S1A). ER orchestrates the proliferation and progression of breast cancer through the binding of estrogen to its estrogen binding site (EBS) within the LBD. This interaction triggers a conformational shift in the protein, leading to dimerization, DNA-binding, and transcriptional activation of genes involved in proliferation, differentiation, and survival [6].

The LBD consists of 12 helices, folding into a globular structure with a pocket for hormone binding, a dimerization interface, and the coregulator interaction site, Activation-Function 2 (AF2) [5] (Supplementary Figure S1B). The AF2 site is a hydrophobic cleft on the surface of the ER-LBD that is distinct from the EBS and plays a central role in coregulator binding and transcriptional activation (Supplementary Figure S1B). The coregulators that bind the AF2 pocket contain at least one LxxLL consensus motif, which forms an α-helix stabilized through hydrophobic interactions within the binding groove. This motif engages with critical residues in the AF2 pocket, specifically lys366 on helix 3 [7, 8] and glu546 on helix 12 [8], where the LxxLL is securely anchored through a charge clamp [9]. When an ER antagonist is bound, H12 occupies the LxxLL-binding site and prevents coactivators from binding [10]. Upon agonist binding to the EBS, the ER undergoes a conformational change, causing H12 to flip away from the binding groove. This exposes the AF2 pocket and the LxxLL-binding groove, allowing interaction with coactivators [10].

The three structural domains of ER—the NTD, DBD, and LBD—have interacting surfaces with other biomolecules, such as proteins and nucleic acids, that can be targeted to control cell proliferation [11, 12]. AF1, located within the NTD, is responsible for ligand-independent activation of transcription. Inhibiting AF1 can therefore reduce cell proliferation in environments where estrogen signaling remains active even at low estrogen levels or when other pathways bypass the need for ligand binding [13]. The role of the DBD is to recognize and bind to specific DNA sequences, such as the canonical estrogen response element (AGGTCAnnnTGACCT) [14]. This interaction drives the expression of ER target genes including those involved in cell proliferation [15]. Targeting this interaction would effectively block ER’s ability to activate transcription, however, this has proven to be difficult due to the highly conserved structure of nuclear receptor DBDs [16–18]. The LBD is the site of estrogen binding and also contains the AF2 pocket for coregulator binding [19].

ER-positive breast cancers are typically treated with hormone therapies that include antiestrogens (such as tamoxifen [20]), aromatase inhibitors (such as letrozole [21], anastrozole [22], or exemestane [23]), and luteinizing hormone-releasing hormone (LHRH) agonists (such as goserelin [24] and leuprolide [25]). These treatments aim to limit estrogen production [26], prevent estrogen binding to the EBS [27], or promote ER degradation [28], thereby inhibiting the growth of cancer cells that rely on estrogen for proliferation. One notable pharmacological agent is tamoxifen, an FDA-approved selective estrogen receptor modulator (SERM), that acts as a competitive antagonist of estradiol, thereby reducing ER transcriptional activity [29]. Another antiestrogen, fulvestrant, was developed by modifying the 7⍺ position of estradiol with a long hydrophobic chain [30]. This modification resulted in an estradiol analog that has a higher affinity for ER comparted to tamoxifen. However, due to its poor solubility, fulvestrant is not orally bioavailable and must be administered via intramuscular injection [31].

Despite their initial efficacy, prolonged use of endocrine therapies typically leads to the development of resistance within 3–5 years in 40% of patients with localized breast cancer and nearly all patients with advanced breast cancer [32, 33]. Among the mechanisms underlying this resistance are mutations in the LBD. Specifically, the Y537S and D538G mutations in helix 12 render ER constitutively active and enhance interactions with coregulators at the AF2 site [34]. These mutations have been shown to contribute to tumor proliferation and reduce the effectiveness of conventional treatments targeting the EBS [34, 35]. Additionally, overexpression of coactivators can lead to resistance to these traditional therapies [36]. Therefore, targeting the AF2 site on ER offers a targeted approach by specifically disrupting the receptor’s ability to recruit coactivators essential for gene activation.

In this study, we conducted an extensive in silico and in vitro screening approach directed at the ER-AF2 site using our recently developed Deep Docking computational method [37]. We have identified 11 promising compounds, including VPC-260724, that show low to mid-range micromolar inhibition of breast cancer cells. This new class of compounds could provide alternative therapeutics for hormone resistant breast cancer patients or be combined with other treatments to enhance efficacy, providing a more comprehensive approach to managing ER-positive cancers.

## Methods

### Preparation of the AF2 site

The crystal structure of the ER-LBD (PDB 3UUD) [38] was selected for virtual screening as it carries the Y537S mutation which stabilizes the active state of ER in the absence of ligands and confers resistance to tamoxifen [39]. Moreover, the AF2 site is occupied by a coactivator peptide (SRC1), and the structure was recently used successfully in a virtual screen of a smaller library [40]. The structure was prepared using Schrödinger Protein Preparation Wizard [41]. Water and other molecules were removed, except for the SRC1 peptide. Preprocessing of structures was carried out with the default parameters of Protein Preparation Wizard (bond order assignment using the CCD database, addition of hydrogens, generation of disulfide bonds, and water molecules removal). Missing side chains were generated with Prime. In the refinement step, PROPKA was used to assign protonation states of protein residues at pH 7.4, and hydrogens of altered species were minimized. The standard minimization procedure of the structure implemented in the Protein Preparation Wizard was also used. The docking grid was centered on the coactivating peptide bound to AF2 and generated in Schrödinger Maestro [42].

### Virtual screening of ZINC15

Deep Docking [37] and Glide SP docking [43] were used to virtually screen the ZINC15 database [43], using default parameters across four iterations [44]. We used the cheminformatics Rdkit [45] package to translate SMILES into Morgan fingerprints of a fixed size of 1,024 bits and a radius of 2, consistent with previous works [44]. Prior to docking, the dominant tautomer and ionization forms of compounds at physiological pH were computed with OpenEye QUACPAC [46] and translated into low-energy 3D conformers with OpenEye Omega [47]. The top scoring compounds were clustered in MOE [48] based on chemical similarity, and docking poses of the best scoring compounds from each cluster were visually inspected to prioritize compounds for testing.

### Chemical and shape similarity search on ZINC20

Compounds selected from the virtual screen were used as templates for ligand-based screening of the ZINC20 database [49]. We selected compounds with a Tanimoto score > 0.5 between their Morgan fingerprints and the query ones. We used the OpenEye ROCS shape matching algorithm [50] to identify additional compounds with 3D conformations similar to the docked poses of the active compounds. We then merged the resulting lists of compounds and docked them into the AF2 site by using Glide SP. We visually inspected the docking poses of the molecules. The 290 well-docked compounds, including compound VPC-260724, which occupies both hydrophobic features at Leu690 and Leu694 of the SRC1 peptide (as shown in Fig. 4A), were selected for testing.

### Cell lines

T47D-KBluc (ER^+^, PR^+^, HER2^−^, G protein-coupled estrogen receptor 1 (GPER)^+^), T47D (ER^+^, PR^+^, HER2^−^, GPER^+^), MCF7 (ER^+^, PR^+^, HER2^−^, GPER^+^), PC3 (ER^−^, PR^−^, HER2^−^, GPER^+^), MDA-MB-231 (ER^−^, PR^−^, HER2^−^, GPER^+^), and MDA-MB-468 (ER^−^, PR^−^, HER2^−^, GPER^+^) cells were purchased from the American Type Culture Collection (ATCC), and PC3m-luc cells were obtained from the Laboratory Corporation of America (Labcorp). All cells were maintained in RPMI 1640 (Gibco, Life Technologies) supplemented with 10% fetal bovine serum (FBS). TamR3 cells, a kind gift from Dr. Euphemia Leung (University of Auckland, New Zealand), were cultured in phenol red-free RPMI 1640 supplemented with 10% charcoal-stripped serum (CSS) and 1 µM tamoxifen. The cells were starved in phenol red-free RPMI 1640 supplemented with 10% CSS, 4.5 g/L glucose (Gibco A24940-01), 1 mM sodium pyruvate (Gibco 11360-070), and 0.2 U/ml insulin (Sigma, I9278). All cells were cultured in a humidified incubator at 37 °C and 5% CO_2_ and routinely checked for mycoplasma contamination.

### Luciferase transcriptional assay

T47D-KBluc cells or PC3m-luc cells were starved for 72 h and then seeded into 96-well plates at 20,000 or 5,000 cells/well, respectively. Cells were treated, 24 h post seeding, with inhibitors at either 50 µM (initial screen) or a 1:2 serial dilution from 50 µM to 0.01 µM (IC_50_ determination) in the presence of 1 nM estradiol (E2). One day post-treatment, the media was removed, and cells were lysed with 65 µl of 1x passive lysis buffer (Promega #E1910). Following two freeze-thaw cycles, 20 µl of lysate were transferred to a white, 96-well flat bottom plate (Corning Life Sciences Cat#3912). 50 µl of luciferase reagent (Promega. Luciferase Assay System E1500) was added to each well, and luminescence was measured using the TECAN M200 Pro plate reader. E2 (1 nM), 4-hydroxytamoxifen (OHT) (4 nM), and DMSO (0.2%) were used as controls for this assay. Due to DMSO toxicity exhibited in cells when exposed to > 1% DMSO, the lowest possible concentration, 0.2%, was used. None of the compounds exhibited precipitation or aggregates at this concentration. E2 alone represented 100% transcriptional activation, while the 0.2% DMSO control represented 0% transcriptional activity. OHT was used as a positive control for an active compound.

### PrestoBlue viability assay

T47D, MCF7, TamR3, and MDA-MB-231 cells were starved for 72 h and then seeded into black clear-bottom 96-well plates (Corning Life Sciences Cat#3904) at 20,000 cells/well (T47D, MCF7, TamR3) or 5,000 cells/well (MDA-MB-231). 24 h later, cells were treated with inhibitors at either 50 µM (initial screen) or a 1:2 serial dilution from 50 to 0.01 µM (IC_50_s) in the presence of 1 nM E2. 20 µl of PrestoBlue Cell Viability Reagent (Thermo Fisher A13262) was added to each well and incubated at 37 °C for 1 h. Fluorescence intensity was measured using the TECAN F500 plate reader with emission and excitation wavelengths of 535 nm and 612 nm, respectively.

### Viability assay in 3D spheroids

MCF7 and T47D cells were seeded into a Corning Costar ultra-low attachment, round-bottom, 96-well plate (Millipore Sigma, CLS7007-24EA) at a density of 2,500 cells/well in starvation media. After 4 days of starvation, the cells were treated with a 1:2 serial dilution of inhibitor from 50 µM to 0.2 µM in the presence of 1 nM estradiol. Images were taken every 6 h using the IncuCyte S3 system and spheroid sizes were determined with the IncuCyte analysis software.

### ER-LBD protein purification

The ER-LBD (residues 298–554) was cloned into pET28a bacterial expression plasmid with an N-terminal His-tag. pET28a-His-ER and pRARE were transformed into *E. coli* (BL21-DE3). Bacteria were grown at 37$\:^\circ\:$C overnight, and then diluted 1:20 the following day in LB media and grown at 37$\:^\circ\:$C until the OD600 reached 0.5. Protein expression was induced with 1 mM IPTG, and 50 µM E2 was added to the media. Cells were then grown for 5 h at 25 °C. Bacterial pellets were collected by centrifugation at 13,000 rpm for 7 min. Pellets were resuspended in lysis buffer (25 mM Tris, pH 8.4, 150 mM NaCl, 20 µM E2, 1 mM DTT, 0.1 mM PMSF, cOmplete protease inhibitor (Roche)) then sonicated and centrifuged to obtain the soluble extracts. ER-LBD was purified using affinity chromatography with Ni-NTA agarose resin. The presence of the ER-LBD in eluted fractions was confirmed by SDS-PAGE and validated by mass spectrometry. The eluted protein was further purified by size exclusion chromatography (s75), and fractions containing the ER-LBD protein were pooled and concentrated using Amicon Ultra centrifugal filters (3 K MWCO).

### Microscale thermophoresis assay (MST)

MST was used to validate the direct binding and measure the binding affinities between the lead compounds and the recombinant ER-LBD. ER-LBD was labeled with red fluorescent dye using a Protein Labeling Kit RED-NHS 2nd Generation (Nanotemper MO-L011) according to the manufacturer’s protocol. The compounds were serially diluted 1:2 in 100% DMSO from 50 to 0.0015 mM, then added to 10 nM of labeled ER protein in the assay buffer (25 mM Tris, pH 8.4, 150 mM NaCl, 20 µM E2, 0.1 mM PMSF, 0.05% Tween20) at final concentrations of 0.03 to 1000 µM. Recombinant proteins in cell free assays are not affected by DMSO concentrations up to 10%. A final concentration of 2% DMSO was chosen for MST as the compounds required this concentration to be soluble in the assay buffer. Following the addition of ER protein, compounds VPC-260709, VPC-260711, VPC-260936, and VPC-260955 exhibited precipitation at 1000 and 500 µM. The corresponding data points were removed from the analysis. Compound VPC-260724 did not exhibit precipitation at any concentration. Each protein/compound mixture was then loaded into Monolith Premium Capillaries (Nanotemper MO-K025). All assays were conducted with medium MST power and 20% excitation power. Data analysis and Kd estimation were performed with the MO Affinity Analysis software from Nanotemper.

### TR-FRET assay

We performed a TR-FRET assay to assess whether compounds displace the AF2 peptide from the ER-AF2 pocket using the LanthaScreen TR-FRET ER alpha Coactivator Assay Kit (Thermo Fisher, A15885). The assay utilizes GST-tagged ER-LBD (final concentration, 7.3 nM), terbium-labeled GST-antibody (final concentration, 5 nM), and a fluorescein-coactivator peptide, PGC1a (final concentration, 250 nM) in the presence of 4 nM E2. The GST-antibody, ER protein and fluorescent peptide mixture was incubated with four concentrations of compound (500 µM, 167 µM, 56 µM, 18.5 µM) for 4 h. The sample was excited at 340 nm and read at 520 nm on a Syngergy Neo2 plate reader (BioTek).

### E2-displacement assay

The E2 displacement assay was performed using the PolarScreen Estrogen Receptor Alpha Competitor Assay, Green (ThermoFisher A15882) according to the manufacturer’s protocol. In brief, full length ERɑ protein was incubated with Fluormone ES2 Green (Fluormone Tracer) in the ES2 screening buffer in a black bottom 384-well plate (Corning 3573) in the presence of either 1, 10, or 50 µM of test compounds. The final concentrations in the assay were 2% DMSO (consistent with the MST assay), 4.5 nM fluormone, and 40 nM ERɑ protein. 20 µl of the mixture were added to each well, with triplicates performed for each condition. The plate was incubated in the dark at room temperature for 4 h before fluorescence polarization was measured using a Synergy Neo2 plate reader (BioTek).

### Proximity ligation assay (PLA)

MCF7 or TamR3 cells were starved for 72 h and then seeded onto PEI coated coverslips in a 6-well plate at 1 × 10^5^ cells/well. For the E2 experiments, the cells were treated the following day with 10 µM of ER-AF2 inhibitor for 24 h, with E2 added 1 h before fixation. For the tamoxifen experiments, 1µM Tamoxifen was added simultaneously with the 10 µM ER-AF2 inhibitor treatment. 24 h post-treatment, cells were fixed in methanol:acetone (3:1). Coverslips were then permeabilized with 0.05% Triton X-100 for 5 minutes at room temperature. The Duolink In Situ Detection Reagents Red kit from Millipore Sigma (DUO92008) was used for blocking and PLA according to the manufacturer’s protocol. Primary antibodies, Estrogen Receptor alpha antibody (F-10) (sc-8002) and Rabbit anti-SRC3 antibody (A300-348 A), were diluted 1:50 in antibody diluent.

### Real-time PCR

MCF7 or TamR3 cells were starved for 72 h then treated with either 1 nM of E2 or 1 µM Tamoxifen and 10 µM of VPC-260724. Total RNA was extracted using TRIzol, 72 h post-treatment. cDNA synthesis was conducted using the Maxima First Strand cDNA Synthesis Kit for RT-qPCR (Thermo K1641) according to the manufacturer’s protocol. RT-PCR was conducted on the QuantStudio 7 Pro instrument and expression fold change was calculated using 2−ΔΔCt method normalized to 18s.

### Nuclear receptor - luciferase transcriptional assay

PC3 cells (androgen receptor (AR)-, glucocorticoid receptor (GR)+, progesterone receptor (PR)-, ER-) were starved for 72 h then seeded into a 96-well plate at a density of 5,000 cells/well. 24 h later, cells were transfected with either ER, AR, GR, or PR and a luciferase plasmid (ERE-luc for ER and ARR3tk-luc for AR, GR, and PR). 24 h post-transfection, cells were simultaneously stimulated with 1 nM E2 for ER, 0.1 nM R1881 for AR, 1 nM Dexamethasone for GR, or 1 nM Levonorgestrel for PR, and treated with 1 or 5 µM of VPC-260724 24 h post-treatment, cells were lysed with 50 µl of passive lysis buffer (Promega #E1910). 20 µl of lysate was transferred to a white flat-bottom 96-well plate (Corning Life Sciences Cat#3912). Luminescence readings were obtained using the TECAN M200 Pro plate reader following the addition of 50 µl of luciferase reagent (Promega Luciferase Assay System E1500).

### Statistical analysis

Statistical analyses for the spheroid assay, TR-FRET, E2 displacement, PLA, RT-PCR, and nuclear receptor luciferase transcriptional assay were performed using GraphPad Prism Version 10.0.2 (GraphPad Software, Inc) with an unpaired t-test (ns, not significant; *, *P* < 0.05; **, *P* < 0.01; ***, *P* < 0.001; ****, *P* < 0.0001).

## Results

### In silico screening and identification of promising actives in cell-based assays used for subsequent similarity search

**Fig. 1:**
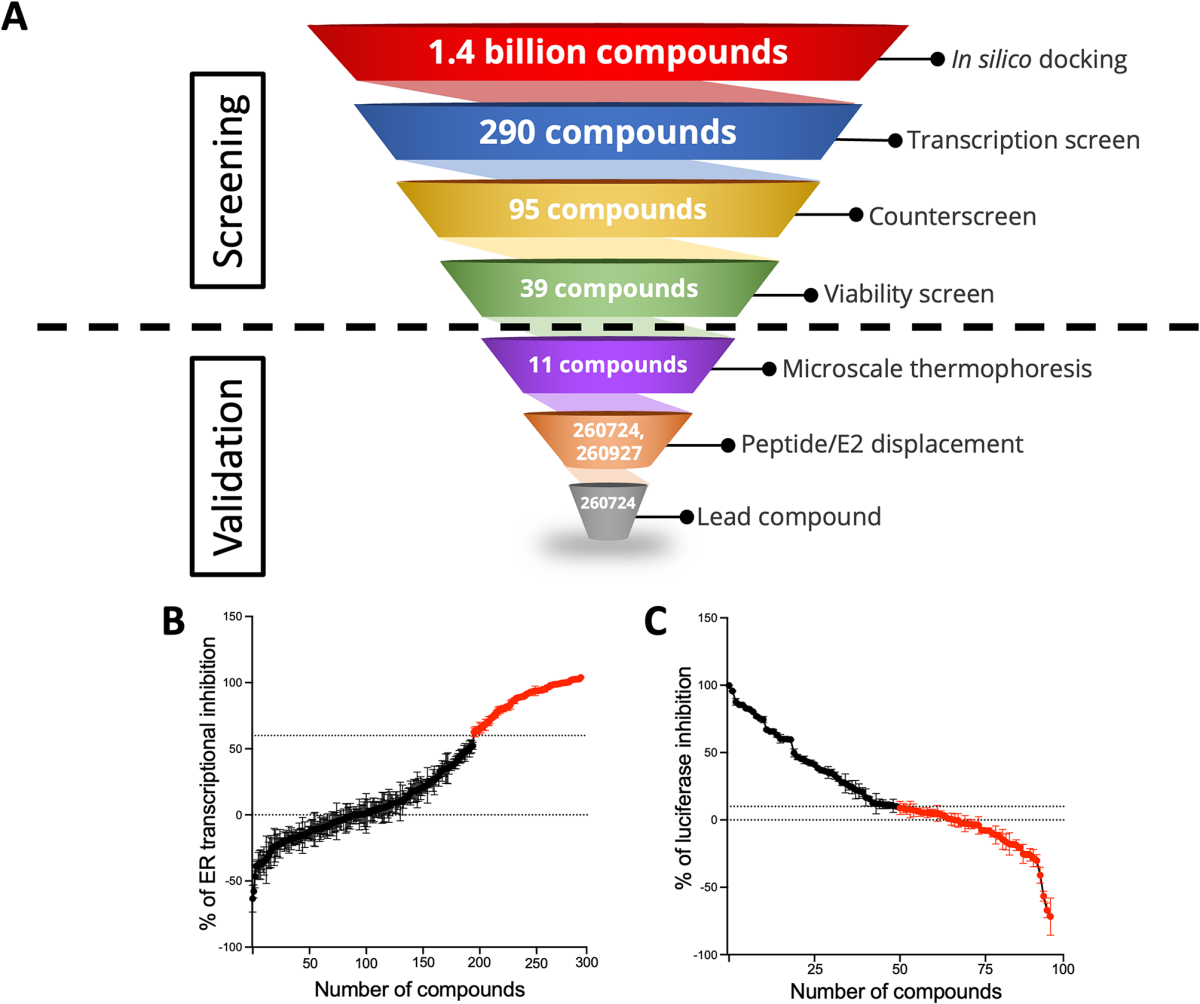
Initial experimental screens in cell-based assays to eliminate inactive compounds. (**A**) Screening pipeline used for development of AF2 inhibitors. (**B**) 290 compounds from in silico docking were evaluated for their inhibition of ER transcriptional inhibition in T47D-KBluc cells at 50 µM of tested compound and 1 nM E2 for 24 h following starvation for 4 days. Compounds presenting ≥ 60% inhibition (highlighted in red) were considered positives. (**C**) The 95 compounds that passed the transcription screen were evaluated for the inhibition of luciferase in ER-negative PC3m-luc cells. The cells were incubated with 50 µM of tested compound and 1 nM E2 for 24 h following starvation for 4 days. 39 compounds (highlighted in red) that showed ≤ 10% luciferase inhibition due to ER-independent effect were considered positive and were retained for further evaluation

We used Deep Docking [51] in combination with Glide Single Precision (SP) [43] to screen the entire ZINC15 database [43]; briefly, Deep Docking is a neural network-based active learning pipeline enabling the identification of top scoring molecules in a library by docking a small fraction of the database. This approach allows for a significant scaling up of virtual screening [44]. The virtual screen reduced the database from 1.36 billion to 9.2 million molecules, which were predicted to have docking scores lower than − 7.14 kcal/mol. This threshold—corresponding to the top 0.01% of scoring molecules in the validation set, (similar to our previous applications of Deep Docking in ultra-large screens [44, 52]), was used to select molecules for subsequent docking into the AF2 site (PDB 3UUD) [38]. After the visual inspection of the resulting docked poses, compounds with incorrect tautomer or protonation states were removed. The remaining compounds had to satisfy the pharmacophore model, which included two hydrophobic features at the Leu690 and Leu694 of the SRC1 peptide. A total of 512 compounds were selected for testing. Compounds were first evaluated in a transcription screen at 10 µM using T47D-KBluc cells, an ER-positive breast cancer cell line that stably expresses a luciferase reporter under the control of three estrogen responsive elements (ERE) upstream of the luciferase gene (Supplementary Figure S2A). Compounds that exhibited more than 40% transcriptional inhibition were tested in a counter screen in ER-negative PC3m-luc cells to determine if they had off-target effects (Supplementary Figure S2B). Next, we evaluated the effect of these compounds at 50 µM on the viability of ER-negative PC3m-luc cells to eliminate toxic compounds (Supplementary Figure S2C). T47D-KBluc cells were used for the transcriptional assay while the viability assay was conducted in T47D cells to assess the effects of the compounds. Additionally, ER negative MDA-MB-468 cells were used in the viability assay to evaluate off-target or non-specific inhibition of cell viability (Supplementary Figure S2E-F). From these screens, four compounds —VPC-260156, VPC-260241, VPC-260263 and VPC-260277 (structures shown in Supplementary Figure S2D)— were identified as promising actives with IC_50_ values in the 5 to 10 µM range. We next performed PLA to assess ability of these compounds to disrupt the interaction between ER and its well-known coactivator, SRC3. This assay allows for the quantification of interactions between two proteins of interest through antibody-based detection methods. All four compounds significantly reduced the number of interactions between ER and SRC3 (Supplementary Figure S2 G-H). We then used the four identified compounds to run a similarity search campaign on ZINC20 [49] (see Methods), that was released during the study. Upon docking, 290 compounds were selected for second round testing, with the goal of identifying robust lead compounds. The in silico-selected 290 compounds were subjected to a screening pipeline detailed in Fig. 1A.

### Cell-based screening and characterization of potential ER-AF2 inhibitors

The 290 compounds were first assessed for their capacity to inhibit ER transcriptional activity in T47D-KBluc cells. We thus treated the cells with 50 µM for 24 h (Fig. 1B and Supplementary Figure S3), and the effect on ER transcription was measured using a reporter assay. We identified 95 positive compounds that exhibited a transcriptional inhibition over 60%. These were further tested for potential off-target effects in ER-negative PC3m-luc cells, which stably and constitutively express luciferase (Fig. 1C and Supplementary Figure S4). Stable expression of the luciferase reporter in the PC3m-luc cells allowed for more consistency between test conditions. We considered molecules with over 10% luciferase inhibition in the counter screen to have off-target effects or non-specific inhibition of the luciferase enzyme and therefore omitted them from further analysis. As a result, we identified 39 compounds with ER-specific inhibition.

### Evaluation of the effect of AF2 inhibitors on cell viability in 2D and 3D models

**Fig. 2:**
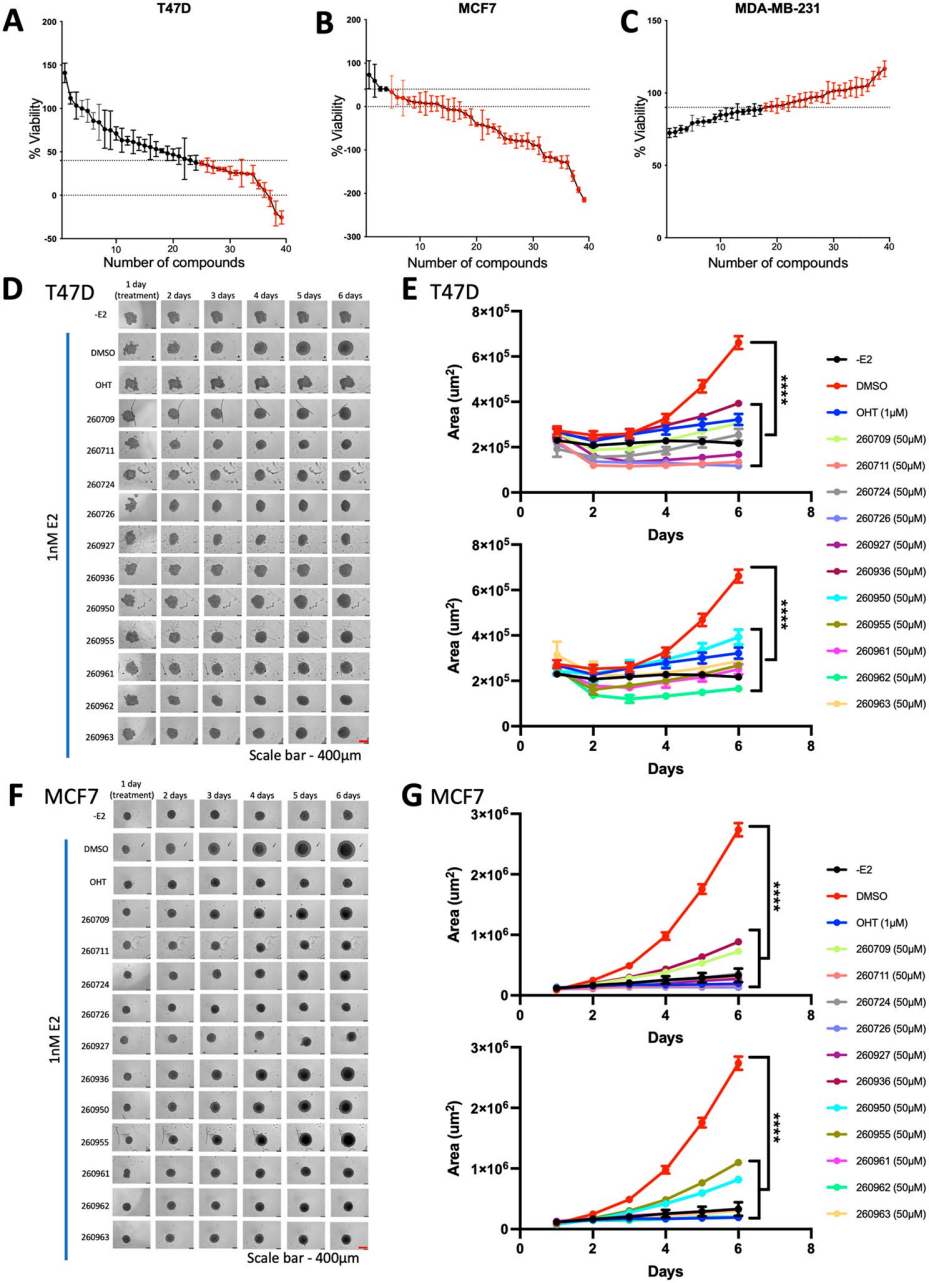
Viability screen for potential AF2 compounds. Viability screen in T47D (**A**), MCF7 (**B**), and MDA-MB-231 (**C**) cells. Positive compounds (highlighted in red) are those that reduced viability in ER-positive cells by more than 60% and caused less than 10% reduction in viability in ER-negative MDA-MB-231 cells. T47D (**D**) and MCF7 (**F**) cells were starved for 3 days, and spheroids were formed in a round-bottom low-binding 96-well plate for 24 h in starvation media. Cells were simultaneously stimulated with 1 nM E2 and treated with wither 50 µM of the studied compound or vehicle (DMSO), then incubated at 37 °C for 5 days. Area of T47D (**E**) and MCF7 (**G**) spheroids plotted over time showed significant reduction of area compared to DMSO control. P values are indicated by stars: ns ≥ 0.05, * 0.01 to 0.05, ** 0.001 to 0.01, *** 0.0001 to 0.001, **** <0.0001

We next evaluated the effect of these 39 molecules on cell viability in three breast cancer models: two ER-positive cells T47D (Fig. 2A) and MCF7 (Fig. 2B) to show the specificity of the compounds to ER, independent of the cellular background, and one ER-negative cell line MDA-MB-231 (Fig. 2C). As described above, we considered compounds to be positive if they reduced the viability of ER-positive cells by at least 60% and did not affect the viability of ER-negative MDA-MB-231 cells by more than 10% (Fig. 2C and Supplementary Figure S5). We selected 11 positive compounds for further characterization by assessing their effects on the viability of 3D breast cancer models using a spheroid assay. This assay determines the ability of compounds to penetrate the 3D mass of cancer cells. To do so, T47D and MCF7 cells were cultured in ultra-low attachment plates to form three-dimensional spheroids. The spheroids were then treated with either vehicle or 50 µM of the studied compounds for 5 days. All 11 compounds exhibited a significant reduction in spheroid size compared to vehicle control (Fig. 2D-G). Thus, these molecules demonstrate selective inhibition of ER-mediated transcription and cell viability in both 2-D and 3-D ER-positive breast cancer models.

### Evaluation of dose response inhibition of ER transcriptional activity and breast cancer cell viability by lead AF2 inhibitors

Following the preliminary screens that tested all compounds at 50 µM, we evaluated the dose-dependent effects of the 11 lead compounds in transcriptional and viability assays (Table 1 and Supplementary Figure S6). For transcription, we used T47D-KBluc cells stably expressing the luciferase reporter, similarly to Fig. 1B, to evaluate the effect of all 11 molecules and to determine their corresponding IC_50_ values. We found that compounds VPC-260711, VPC-260724, and VPC-260962 showed low micromolar inhibition of ER transcription in T47D-KBluc cells with IC_50_ values of 6.4, 5.7, and 10.8 µM, respectively. To assess the effects of the 11 compounds on cell viability, we examined the dose-dependent inhibition of four cell lines: ER-positive T47D and MCF7, the ER-positive and tamoxifen-resistant TamR3 cell line, and ER-negative MDA-MB-231 cells. VPC-260711, VPC-260724, and VPC-260962 reduced viability in all three ER-positive cell lines, with low micromolar IC_50_ values of 5.4, 7.4 and 8.9 µM, respectively, in T47D cells. Notably, there was minimal impact on MDA-MB-231 cells, suggesting that these compounds have an ER pathway specific mechanism of action.

**Table 1:**
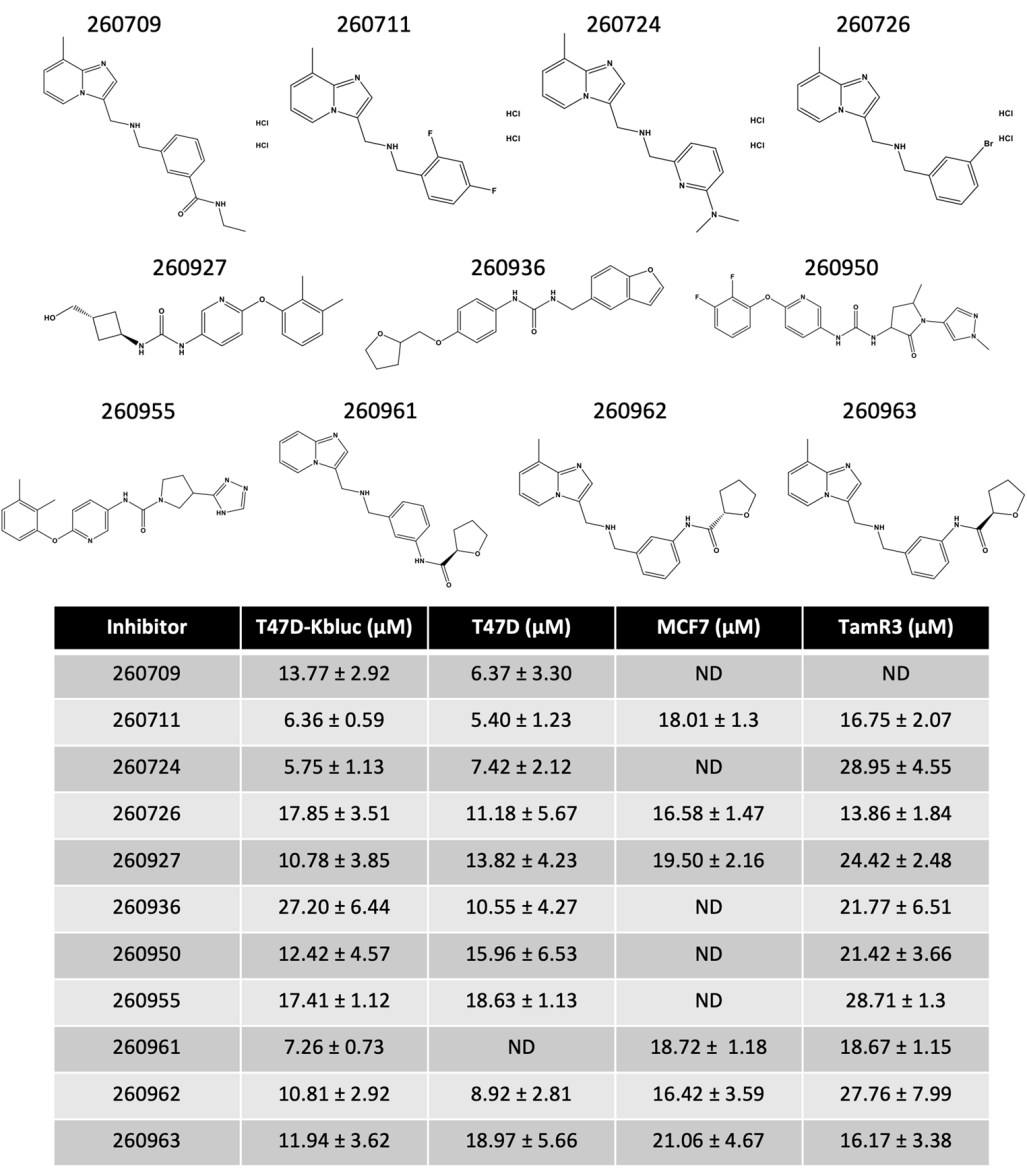
Structure and IC_50_ values of current lead compounds obtained after initial transcription and viability screen. ± refers to the standard deviation across 3 biological replicates; ND – not determined

### Direct binding evaluation of lead inhibitors to AF2 site

**Fig. 3:**
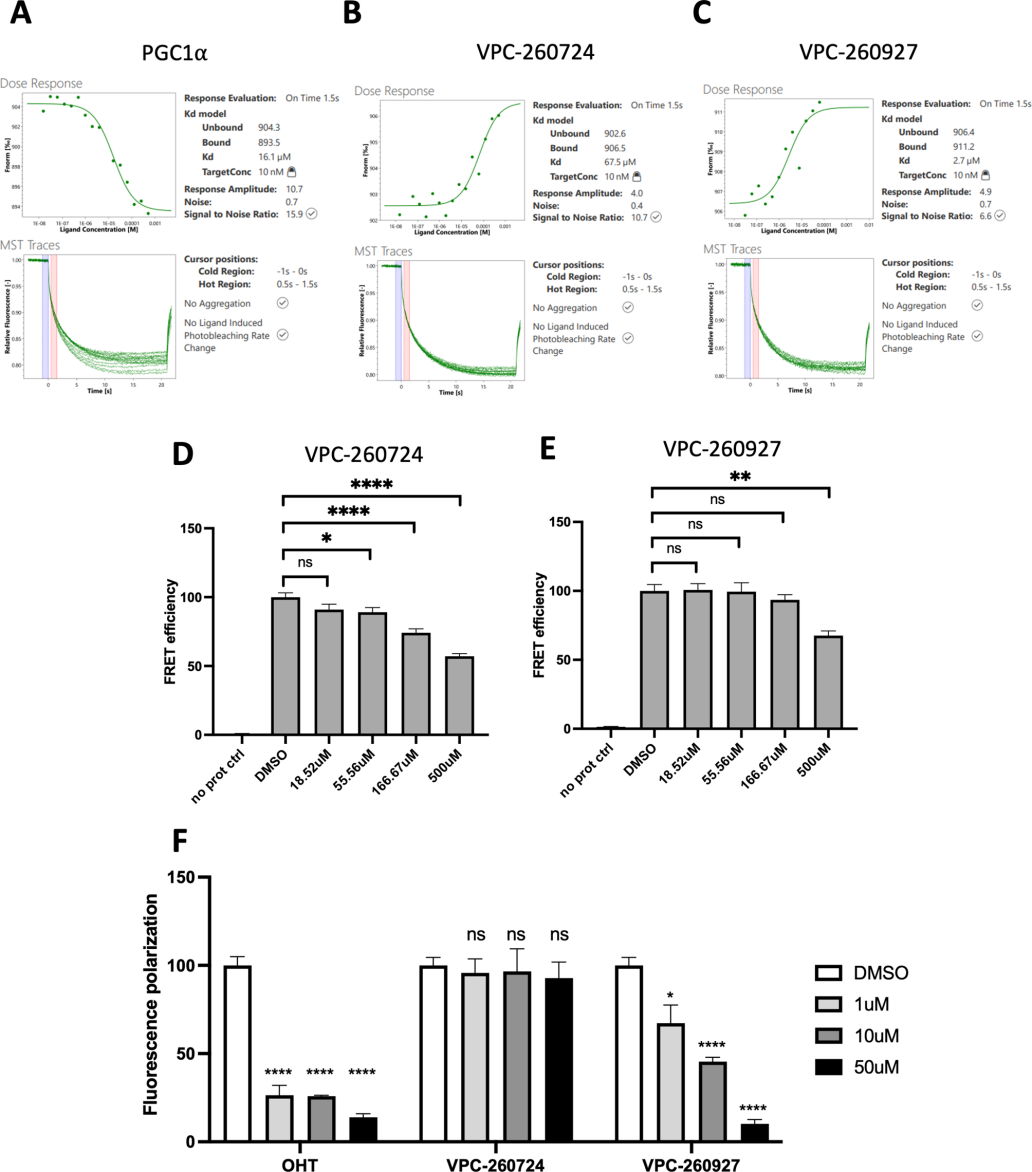
Cell-free characterization of lead compounds. Representative dose-dependent binding curves of the control peptide PGC1⍺ (**A**), VPC-260724 (**B**), and VPC-260927 (**C**) to recombinant ER-LBD, as measured by microscale thermophoresis (MST). The reported K_d_ values were averaged from three independent assays (*n* = 3). Peptide displacement from AF2 site as determined by TR-FRET assay for lead compounds VPC-260724 (**D**) and VPC-260927 (**E**). (**F**) E2 displacement from EBS fluorescence polarization of VPC-260724 and VPC − 260927 with OHT control. P values are indicated by stars: ns ≥ 0.05, * 0.01 to 0.05, ** 0.001 to 0.01, *** 0.0001 to 0.001, **** <0.0001

To validate the direct binding of our identified molecules to ER-LBD, we performed MST analysis. ER-LBD (residues 298–554) was expressed, purified, then labelled with a red fluorescent tag using Protein Labeling Kit RED-NHS 2nd Generation from NanoTemper. We compared the movement of fluorescently-labeled ER-LBD, in a temperature gradient induced by an infrared laser, alone or when incubated with increasing concentrations of our 11 lead molecules ranging from 0.03 to 1000 µM. The shift of the fluorescently labelled protein was plotted against ligand concentration to calculate binding affinities (K_d_) of the molecules. To validate the folding of our recombinant ER-LBD protein, we used as positive control the coactivator peptide PGC1ɑ and obtained a K_d_ of 16.6 ± 2.4 µM (Fig. 3A). Among the previously selected 11 compounds, we found that VPC-260724 and VPC-260927 demonstrated a dose-dependent shift of thermophoresis, with estimated binding affinities of 85.2 ± 32.7 µM and 13.2 ± 10.5 µM, respectively (Fig. 3B-C), confirming their direct binding to the ER-LBD. All other compounds did not exhibit direct binding to ER-LBD (Supplementary Figure S7).

### Coactivator peptide and E2 displacement of lead compounds

Next, we evaluated the ability of VPC-260724 and VPC-260927 to displace a coactivator peptide from the AF2 pocket using TR-FRET (Fig. 3D-E). Due to the availability of a commercial kit, the peptide used was derived from coactivator PGC1⍺. In this assay, recombinant ER protein tagged with GST was incubated with an anti-GST terbium conjugated antibody (donor, excitation at 340 nm), fluorescein-labeled PGC1⍺ peptide (acceptor, emission at 520 nm), and varying concentrations of the tested compound. In the absence of compounds, the interaction between ER and PGC1⍺ results in fluorescence emission at 520 nm upon excitation at 340 nm. When the studied compounds were added, a decrease in fluorescence readings at 520 nm indicated reduced interaction between the peptide and ER, demonstrating the ability of compounds to displace the peptide from the AF2 site. At 50 µM, VPC-260724 was capable of lowering the FRET signal, while a reduction was only observed at 500 µM for VPC-260927. Peptide displacement from the AF2 site could also be due to an indirect effect of the compound binding to the EBS. Therefore, we assessed whether VPC-260724 and VPC-260927 bind to the EBS using an E2 displacement assay. This assay utilizes a fluorescent E2 (fluormone) that exhibits high fluorescence polarization when bound to ER-LBD. Compounds that bind to the EBS and displace the fluormone from the site would result in a reduction of the fluorescence polarization signal. We found that VPC-260927 displaces the fluormone at 1, 10 and 50 µM, indicating its binding to the EBS while VPC-260724 did not displace the fluormone at any of the tested concentrations, confirming its binding to AF2 site (Fig. 3F).

#### Characterization of the lead AF2 inhibitor VPC-260724

From the results described above, VPC-260724 emerged as a promising ER-AF2 inhibitor. This molecule demonstrated inhibition of ER transcriptional activity and reduced viability of ER positive breast cancer models, including Tamoxifen-resistant TamR3 cells (Table 1). Importantly, VPC-260724 also exhibited direct binding to ER-LBD, displacement of coactivator peptide from AF2 site, without displacing E2 from the EBS.

**Fig. 4:**
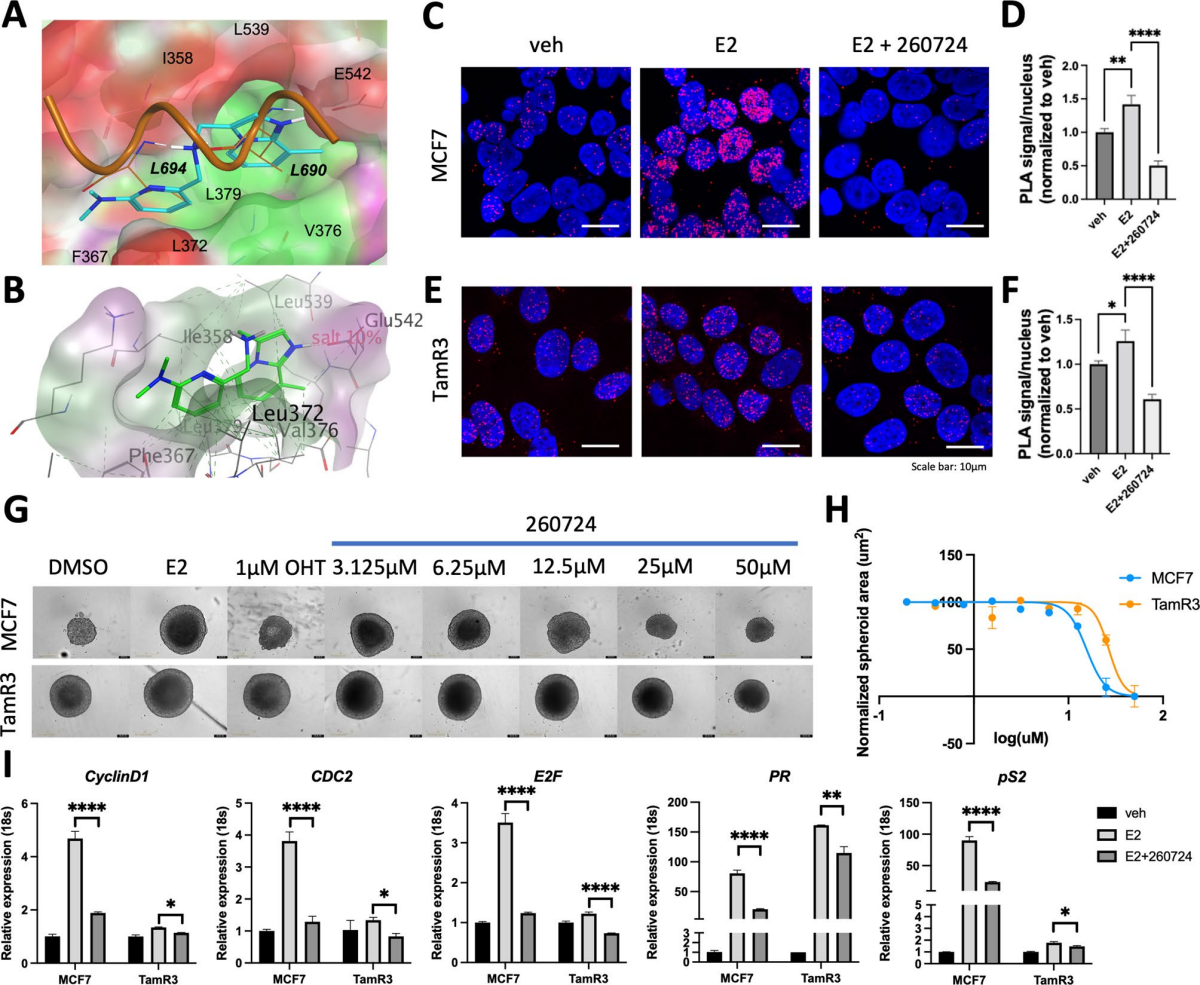
Activity profile of VPC-260724. (**A**) Binding pose of VPC-260724 (cyan) in the AF2 site, predicted by computational docking. The compound occupies the hydrophobic cavity with the pyridine and the imidazopyridine moieties mimicking L694 and L690 residues (in bold italic) of the AF2-binding coactivator and interacting with hydrophobic and aromatic residues lining the AF2 binding site. Green = hydrophobic surfaces, purple = polar surfaces, red = solvent exposed. (**B**) A salt bridge is established between the imidazopyridine group and the side chain of Glu542 of the AF2 site. Proximity ligation assay to determine the effect of lead compound VPC-260724 on the interaction between ER and coactivator SRC3 in MCF7 (**C**) and TamR3 (**E**) cells. Interactions between ER and SRC3 are represented by the red PLA signals. 10 µM treatment of VPC-260724 for 48 h significantly reduced the number of PLA signals per nuclei in both MCF7 (**D**) and TamR3 (**F**) cells following starvation for 4 days and stimulation with 1 nM E2. (**G**) Dose response effect of lead compound VPC-260724 on area of MCF7 and TamR3 spheroids. Cells were starved for 3 days, and spheroids were formed in a round-bottom low-binding 96-well plate for 24 h in starvation media before treatment with a serial 1:2 dilution of VPC-260724 starting at 50 µM. (**H**) Quantification of the dose-dependent inhibitory effect of VPC-260724 on the size of spheroids. (**I**) qPCR showing reduction of ER-target genes mRNA levels. Treatment of MCF7 and TamR3 cells by 10 µM VPC-260724 for 72 h reduced mRNA levels of ER targets: CyclinD1, CDC2, E2F, PR and pS2. P values are indicated by stars: ns ≥ 0.05, * 0.01 to 0.05, ** 0.001 to 0.01, *** 0.0001 to 0.001, **** <0.0001

To characterize the molecular interaction between ER-LBD and VPC-260724, we used Molecular Operating Environment (MOE) [48] to evaluate the binding pose of VPC-260724 within the AF2 pocket. We found that VPC-260724 fills the entire AF2 groove and forms hydrophobic interactions with Ile358, Phe367, Leu372, Ile358, Val376, Leu379 and Leu539. This compound also forms a salt bridge with Glu542 side chain (Fig. 4A and B).

**Fig. 5:**
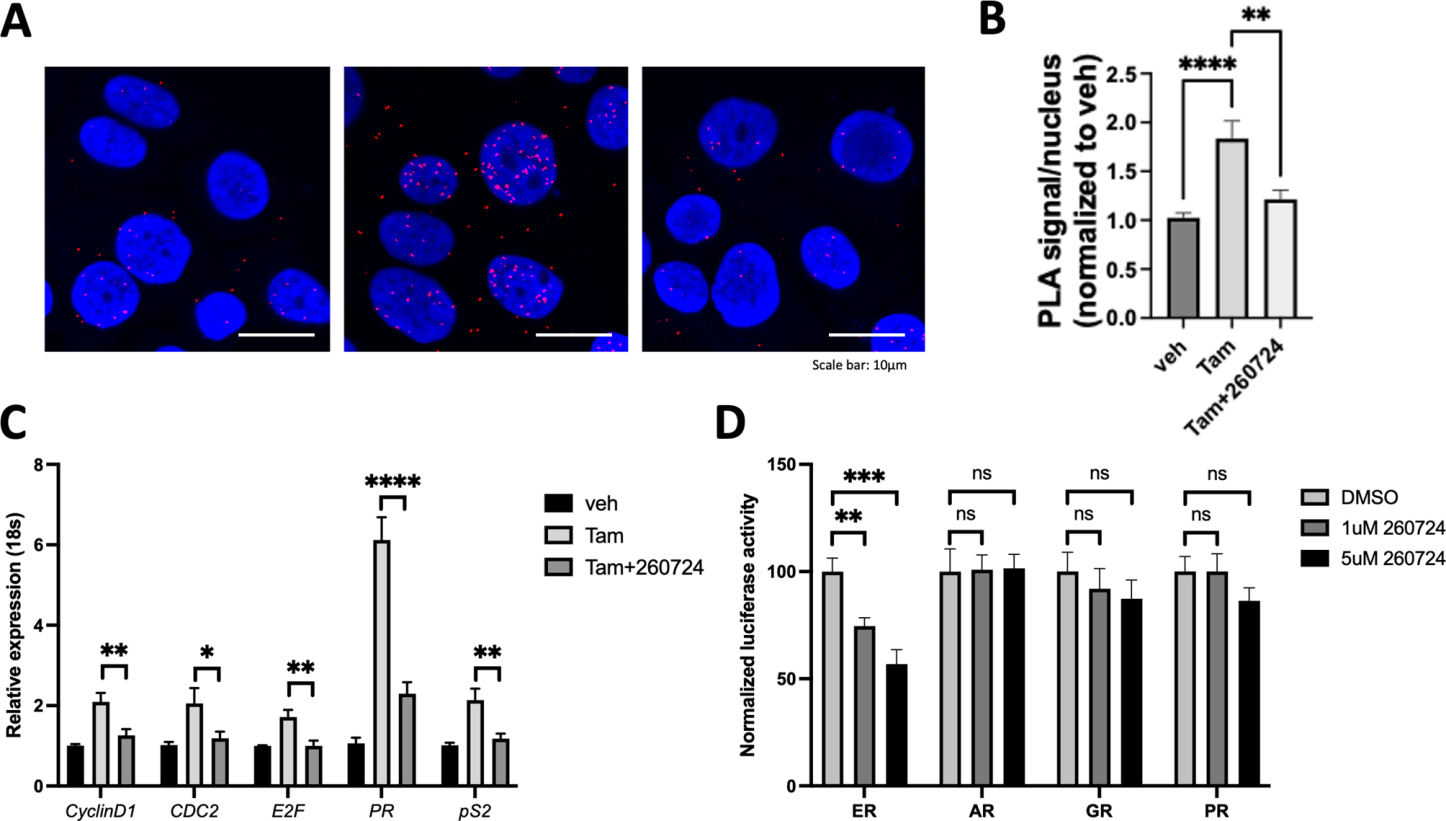
Profiling the activity of VPC-260724. (**A**) Proximity ligation assay to determine the effect of lead compound VPC-260724 on the interaction between ER and coactivator SRC3 in TamR3 cells. The cells were starved in CSS media without Tamoxifen for 4 days before treated with either 1 µM Tamoxifen alone or with 10 µM VPC-260724 and 1 µM Tamoxifen simultaneously. Interactions between ER and SRC3 are represented by the red PLA signals. (**B**) 10µM treatment of VPC-260724 significantly reduced the number of PLA signals per nuclei. (**C**) qPCR showing reduction of ER-target genes cyclinD1, CDC2, E2F, PR and pS2 mRNA levels after 72 h of 10 µM treatment of VPC-260724 in the presence of 1 µM Tamoxifen. (**D**) VPC-260724 is specific for ER, tested at indicated concentrations in luciferase assay transiently transfecting ER, AR, GR and PR with a luciferase reporter plasmid. P values are indicated by stars: ns ≥ 0.05, * 0.01 to 0.05, ** 0.001 to 0.01, *** 0.0001 to 0.001, **** <0.0001

While we have observed disruption of the interaction between the ER-LBD and its coactivator in vitro in the presence of VPC-260724, we aim to confirm this effect in a cellular context using PLA. We thus assessed the effect of this compound on the interaction between full-length ER and its well-known coactivator SRC3 in MCF7 and TamR3 cells (Fig. 4C and E). Due to the availability of a highly specific antibody compared to SRC1 and PGC1⍺, SRC3 was chosen for this assay. Upon E2 stimulation, the number of interactions between ER and SRC3 increased. However, treatment with 10 µM of VPC-260724 for 48 h significantly reduced ER-SRC3 interactions in both MCF7 and TamR3 cells (Fig. 4D and F). We also evaluated how VPC-260724 affected the interactions between SRC3 and two of the most common ER mutants that are resistant to antiestrogens, ER-Y537S and ER-D538G [34]. Excitingly VPC-260724 inhibited the interaction between SRC3 and both of the clinically relevant ER mutants (Supplementary Figure S8A and S8B). Additionally, we tested VPC-260724 on a clinically relevant fulvestrant mutant, D538G/F404L, and found that while fulvestrant increased the transcriptional activity of the mutant ER, VPC-260724 significantly reduced the transcriptional activity compared to DMSO (Supplementary Figure S9). We also evaluated the effect of this compound on ER-SRC3 interactions in a tamoxifen resistance setting. We found that 1 µM Tamoxifen increased ER-SRC3 interaction in TamR3 cells; however, treatment with 10 µM VPC-260724 reduced this interaction (Fig. 5A-B).

A dose-dependent evaluation of VPC-260724 on the growth of MCF7, and TamR3 spheroids resulted in IC_50_ values of 15.6 ± 1.4 µM and 27.0 ± 3.2 µM, respectively when area of each spheroid was plotted as function of VPC-260724 concentration (Fig. 4G and H). Furthermore, mRNA levels of ER target genes (*cyclinD1* [53, 54], *CDC2* [54], *E2F* [54], *PR* [54], and *pS2* [55]) in MCF7 and TamR3 cells presented a significant decrease upon treatment with 10 µM VPC-260724 for 72 h, following E2 stimulation (Fig. 4I). Consistent with the PLA results, TamR3 cells treated with 1 µM Tamoxifen showed increased target gene expression, which was markedly reduced by the combined treatment with addition of 10 µM of VPC-260724 (Fig. 5C).

Additionally, we evaluated the selectivity of VPC-260724 towards ER. We thus investigated whether this compound affects the transcriptional activity of other steroid hormone receptors: the AR, PR, and GR. PC3 cells were transfected with either ER, AR, GR, or PR and their transcriptional activities were measured using luciferase reporters under the control of an ERE for ER and ARR3tk [56] for AR, GR, and PR. While VPC-260724 showed a dose-dependent inhibition of ER, it did not significantly impact the transcriptional activities of AR, GR, or PR (Fig. 5D), confirming its specificity of action.

## Discussion

Up to 80% of breast cancers are positive for the ER and are typically treated with SERMs, such as tamoxifen. Unfortunately, the majority of patients will develop resistance to tamoxifen over the course of their treatment period. Many mechanisms underlie treatment resistance such as loss of ER expression, mutations in the LBD, or altered expression of ER coregulators that bind to and activate ER transcriptional activity [57]. To overcome LBD mutations and coregulator overexpression resistance mechanisms, we proposed to target the AF2 pocket, a hydrophobic groove located in the ligand binding domain of ER and is one of the main sites for coregulator binding. As resistance mutations typically occur in and around the EBS and as majority of ER transcriptional activity is mediated by AF2, successfully inhibiting this site and coregulator recruitment could provide a novel mechanism for inhibition of the receptor.

Our work utilizes a virtual screening approach where through a combination of Deep Docking and Glide SP, over a billion compounds were screened in silico, making this the largest library of compounds virtually assessed for ER-AF2 binding so far. Screening at a 100-fold acceleration compared to traditional methods allowed the classification of potential drug candidates more efficiently, without compromising accuracy [44]. This comprehensive approach identified 290 compounds as potential AF2 inhibitors. The compounds were first put through a screening pipeline where only the ones that passed each screen would move on to the next stage. We used a concentration of 50 µM for all initial screens being the highest concentration we could obtain due to limitations in solubility of the compounds in the 0.2% DMSO that was safe to use in cell-based assays. We first ran all compounds through a transcription screen where we used a cut-off threshold of 60% inhibition of ER transcriptional activity. Although this threshold is relatively low, we did not want to eliminate any compound that may not have performed well in this assay but would have higher efficacy in other assays. A 60% threshold resulted in approximately one third of the compounds moving on to the next screening step. We next evaluated the off-target effect of the selected molecules in counter screen that allowed us to exclude the compounds acting on the luciferase enzyme itself or through ER-independent mechanisms. We next performed viability screens, where we adopted a 60% inhibition in the viability of breast cancer models as a cut-off for positive molecules. After narrowing down the initial list of 290 compounds to 11 compounds, these hits were evaluated for their effects on growth and proliferation of breast cancer cells in 3D culture. All 11 compounds showed significant reduction in spheroid growth. Assessing viability in 3D culture provided a more accurate representation of real cell environments as they mimic solid tumors more accurately and provides insights into the penetration and uptake ability of the compounds.

The potency of the 11 lead compounds was then evaluated by estimating the IC_50_ values of the studied molecules in ER transcriptional and viability assays of multiple cell models. While IC_50_ values predominantly fell in the low micromolar range, further Structure-Activity Relationship (SAR) analyses will be necessary to enhance compound efficacy. To validate direct biomolecular interactions between our lead compounds and ER protein, we utilized microscale thermophoresis which detects the movement of fluorescently labelled protein alone or when in complex with a compound across a temperature gradient. Of the top 11 compounds, two compounds, VPC-260724 and VPC-260927 showed direct binding to purified ER-LBD protein with kds of 85.2 ± 32.7 µM and 13.2 ± 10.5 µM, respectively. Importantly, TR-FRET analysis established an AF2-specific mode of action for VPC-260724 by competing with the PGC1α coactivator peptide and not displacing the estrogen from the EBS. In contrast, VPC-260927 was an EBS binder as it displaced E2 from this pocket.

Although peptide displacement and direct binding assays can provide insightful information on the binding mode of a compound, solving the crystal structure of a small molecule-bound protein would be invaluable in confirming the binding profile. While many groups have shown crystal structures of peptide bound to ER-AF2, obtaining crystals of ER-LBD with small molecules bound to AF2 pocket remains challenging. From the predicted docking pose, VPC-260724 appears to span the entire AF2 hydrophobic cavity and mimic key interactions observed for the coactivator-AF2 complex (PDB 3UUD) (Fig. 4A and Supplementary Figure S1B).

In addition to direct binding experiments, our work incorporated the evaluation of inhibitors in a 3D cell model setting with the implementation of a spheroid assay – an avenue not explored in previous ER-AF2 inhibitor characterization. Lead compound VPC-260724 exhibited a dose-dependent effect on transcription in T47D-KBluc cells as well as on proliferation in T47D, MCF7 and TamR3 in both 2D cell culture and 3D spheroids. The compound did not affect the viability of triple-negative MDA-MB-231 cells. This highlights its ER-mediated specific inhibitory activity, while PLA verified the ability of VPC-260724 to disrupt the interaction between ER and known coactivator SRC3. This ER-directed activity was further confirmed through the downregulation of ERα target genes, *CCND1*, *CDC2*, *E2F*, *PR*, and *pS2* in MCF7 and TamR3 cells. VPC-260724 was also evaluated on the two most common ER mutations, Y537S and D538G, shown to confer endocrine resistance by stabilizing the AF2 pocket even in the absence of E2, as well as the fulvestrant resistant mutant, D538G/F404L. The compound significantly reduced the interactions between each ER, Y537S and D538G, and SRC3 (Supplementary Figure S8), and diminished the transcriptional activity of the D538G/F404L mutant (Supplementary Figure S9).

The current gold standard treatment of ER-positive breast cancer is tamoxifen which competes with E2 from binding to ER at the EBS [58]. While an additional binding site has been identified for ERβ near the AF2 site [59], structural data and our E2 displacement assays do not support the same model for ER⍺ (Fig. 4F). Despite the effectiveness of tamoxifen, many patients with primary breast cancer, and the majority of those with advanced breast cancer, eventually develop resistance to the treatment. To evaluate the performance of VPC-260724 in such scenarios, TamR3 cells treated with tamoxifen was assessed in PLA and qPCR. An increase of ER-SRC3 interactions was observed after tamoxifen treatment which was significantly reduced when the cells were treated with Tamoxifen in combination with VPC-260724. This trend was also observed in target gene expression levels where the combination lowered the relative expression of target genes compared to tamoxifen treatment alone. Altogether, VPC-260724 represents a promising lead compound, which with further optimization can lead to the future development of novel treatment modalities for ER-positive breast cancers, both in treatment–naïve patients and patients that have developed SERM resistance.

Given the structural similarities in the LBDs of steroid receptors within this family of proteins, it was vital to determine the selectivity of VPC-260724 to ER and eliminate the possibility of inhibiting other steroid receptors. To discern this specificity, we conducted an analysis through the reduction of luciferase activity, where PC3 cells treated with VPC-260724 demonstrated a marked decrease in transcriptional activity only when transfected with pcDNA-ER and ERE-luc. PC3 cells transfected with AR, PR, or GR along with a luciferase reporter did not show any significant reduction of transcriptional activity.

Recent works in other research groups have led to the identification of additional AF2 inhibitors. Many of these compounds, such as biphenyl proteomimetics [60, 61] and ERXs [62], were characterized based on their ability to inhibit ER transcriptional activity and displace a coactivator peptide from AF2 site. These evaluations were conducted through various assays, including mammalian two-hybrid assays [60], TR-FRET [61, 63], or pull down assays [62]. VPC-260724 exhibited comparable IC_50_ values in the low micromolar range. While these compounds showed promising results in specific assays, it is important to note that the characterization of most of these inhibitors (with the exception of ERX-11 [62]) did not include direct binding experiments to confirm their interaction with ER. This limitation raises questions about the specificity and mechanism of action of these compounds. In contrast, our study not only assessed the inhibitory activity of VPC-260724 in ER transcriptional activity but underwent a more comprehensive evaluation of the molecules across multiple assays, increasing the confidence in our lead compound and delineating its mechanism of action. Thus, the backbone structure of VPC-260724 can potentially be a great privileged structure for future lead optimization of ER-AF2 directed inhibitors.

## Conclusions

In summary, 290 compounds were identified in silico and evaluated for their ability to block ER activity and inhibit breast cancer cell viability. Lead compound VPC-260724 targets the ER using a different mechanism and at a different site from that of tamoxifen by binding to the AF2 pocket instead of the EBS. This presents a novel mechanism for ER inhibition, providing the potential for a synergistic action with existing treatments, thereby amplifying inhibitory efficacy for the treatment of ER-driven BCa and offering a novel therapeutic route for patients that have already developed resistance to current treatments.

## Electronic supplementary material

Below is the link to the electronic supplementary material.

Supplementary Material 1: Supplementary Figure S1. Estrogen receptor structure. (A) Structural organization of ERα. (B) ER ligand binding domain (PDB: 4J24) with estradiol (purple) bound to the Estrogen Binding site and SRC3 peptide (yellow) bound to the Activation Function 2 site.

Supplementary Material 2: Supplementary Figure S2. Cell-based data used for in silico similarity searches. (A) The percentage of ER transcriptional inhibition after treatment of T47D-kbluc cells with 10 µM of each of the 512 compounds, identified through in silico docking. (B) Counter screen to determine the effect of the compounds on inhibition of luciferase in ER-negative PC3m-luc cells. (C) Viability of current hits in ER-negative PC3m-luc cells to eliminate toxic compounds. (D) Structures of the four selected chemotypes, VPC-260156, VPC-260241, VPC-260263, VPC-260277, highlighted in red in panels A-C. (E) Dose-response inhibition of transcriptional activity measured with luciferase reporter assay in T47D-KBluc cells following treatment of tested compounds for 24 h. (F) Dose-response inhibition of cell viability measured PrestoBlue in T47D and ER-negative MDA-MB-468 cells following treatment of tested compounds for 72 h. (G) PLA showing interaction of ER and SRC3 in T47D cells with quantification of the corresponding PLA signal/nuclei (H). P values are indicated by stars: ns ≥ 0.05, * 0.01 to 0.05, ** 0.001 to 0.01, *** 0.0001 to 0.001, **** <0.0001.

Supplementary Material 3: Supplementary Figure S3. Screening ER inhibitors using a Luciferase reporter-based transcriptional assay. T47D-KBluc cells were treated with 50 µM inhibitor in the presence of 1 nM E2. Luminescence was read after 24 h. Compounds exhibiting >60% inhibition of ERα transcriptional activity were retained as positives for further evaluation.

Supplementary Material 4: Supplementary Figure S4. ER-independent inhibition of luciferase enzyme in PC3m-luc cells. To determine if the compounds affected the luciferase enzyme itself or if there were any off-target effects, PC3m-luc cells were treated with 50 µM of tested compounds and luminescence was read after 24 h. Compounds that inhibited luciferase in these cells are acting through a different pathway from ER. Those that exhibited <10% off-target effect was selected for the next screening assay. 38 compounds passed the counter screen.

Supplementary Material 5: Supplementary Figure S5. Effect of ER inhibitors on the viability of BCa models. T47D, MCF7, and MDA-MB-231 cells were starved for 4 days and treated with 1 nM E2 and 50 µM of compounds for 72 h. PrestoBlue viability assay determined effect of compounds on viability. Compounds that exhibit inhibitory effect on growth of the ER-positive T47D and MCF7 cells (>60%) and minimal effect on the ER-negative MDA-MB-231 cells (<10%) passed this screen. 11 compounds met this criteria.

Supplementary Material 6: Supplementary Figure S6. Evaluation of dose response inhibition of ER transcriptional activity and BCa cell viability by lead AF2 compounds. IC50s of lead AF2 compounds in T47D-KBluc, T47D, MCF7, TamR3, and MDA-MB-231 cells were measured by luciferase reporter-based assay for transcription and PrestoBlue assay for viability. Cells were starved for 4 days following 24 h and 72 h treatment for transcriptional and viability inhibition, respectively.

Supplementary Material 7: Supplementary Figure S7. Microscale thermophoresis curves for inactive compounds. Dose-response curves for direct binding of tested compounds with purified ER-LBD measured by MST. 1:2 serially diluted concentrations of lead compounds starting at 1 mM were tested on the movement of fluorescently labeled ER-LBD in a temperature gradient. Compounds shown here did not exhibit direct binding.

Supplementary Material 8: Supplementary Figure S8. Effect of VPC-260724 on clinically relevant ER mutants. (A) Proximity ligation assay with either ER-WT or ER mutants, Y537S and D538G, and SRC3 treated with 10 µM VPC-260724 in the presence or absence of 1 nM E2. (B) Quantification of ER-WT/mutant – SRC3 interactions as PLA signal/nucleus normalized to vehicle treatment. P values are indicated by stars: ns ≥ 0.05, * 0.01 to 0.05, ** 0.001 to 0.01, *** 0.0001 to 0.001, **** <0.0001.

Supplementary Material 9: Supplementary Figure S9. Effect of VPC-260724 on fulvestrant resistant mutant ER D538G/F404L. (A) IC50 of Fulvestrant and VPC-260724 in TamR3 cells were measured by luciferase reporter-based assay for transcription. (B) Effect of VPC-260724 and fulvestrant on transcriptional activity of ER D538/F404L mutant. P values are indicated by stars: ns ≥ 0.05, * 0.01 to 0.05, ** 0.001 to 0.01, *** 0.0001 to 0.001, **** <0.0001.

## Data Availability

No datasets were generated or analysed during the current study.

## Abbreviations

AF2: Activation-function 2
AR: Androgen receptor
DBD: DNA-binding domain
E2: Estradiol
EBS: Estrogen binding site
ER: Estrogen receptor
ERE: Estroen response element
GR: Glucocorticoid receptor
LBD: Ligand-binding domain
MST: Micrscale thermophoresis
PLA: Proximity ligation assay
PR: Progesterone receptor
SAR: Structure activity relationship
TR-FRET: Time-resolved fluorescence energy transfer
