# Supplementary figures and images for: Characterization of novel small molecule inhibitors of estrogen receptor-activation function 2 (ER-AF2)
Source: Breast Cancer Res. 2024 Nov 26;26:168. doi: 10.1186/s13058-024-01926-2 (PMC11590367; doi:10.1186/s13058-024-01926-2)

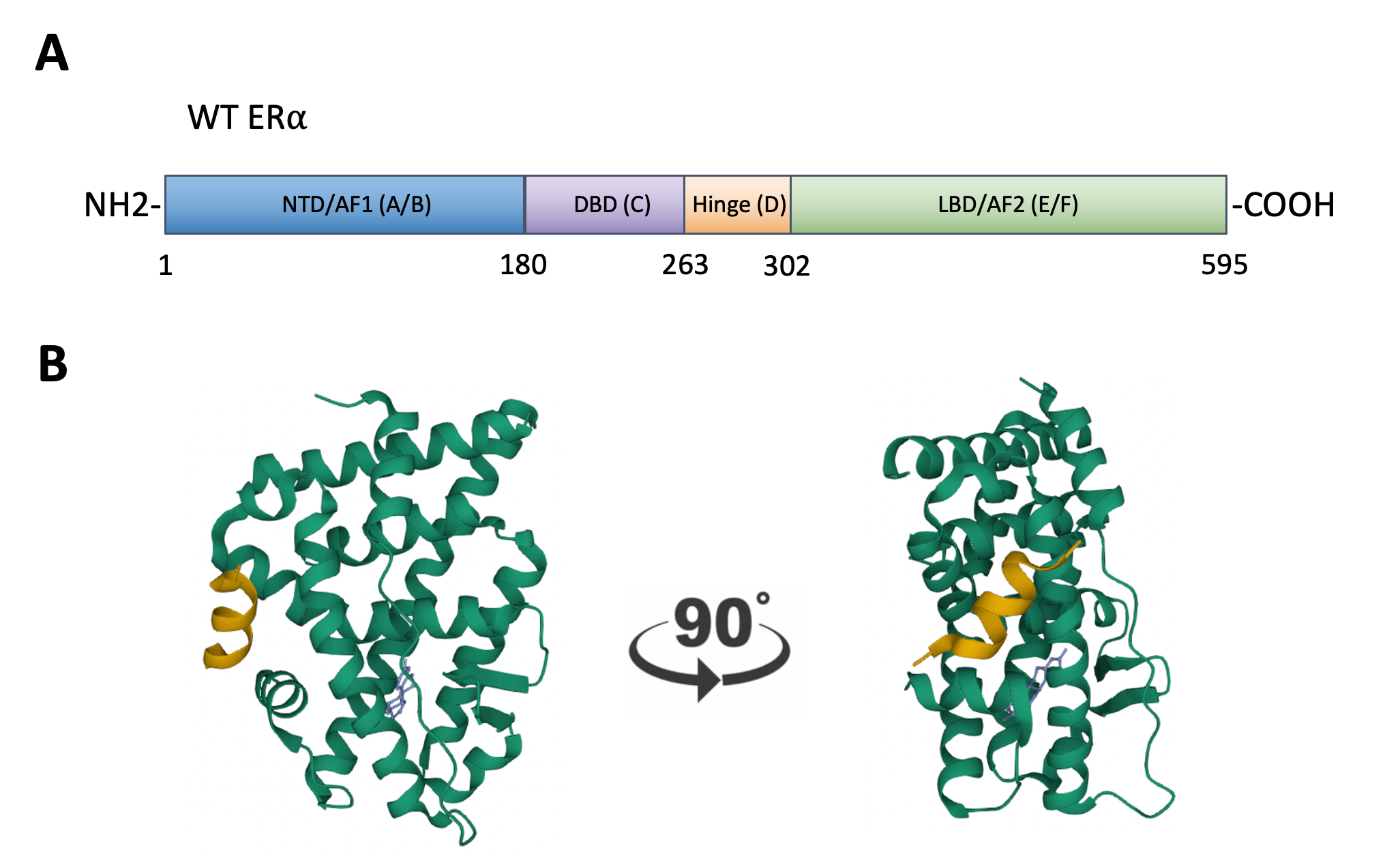

Supplement: Supplementary file 1 — Supplementary Material 1: Supplementary Figure S1. Estrogen receptor structure. (A) Structural organization of ERα. (B) ER ligand binding domain (PDB: 4J24) with estradiol (purple) bound to the Estrogen Binding site and SRC3 peptide (yellow) bound to the Activation Function 2 site. [file 13058_2024_1926_MOESM1_ESM.png]

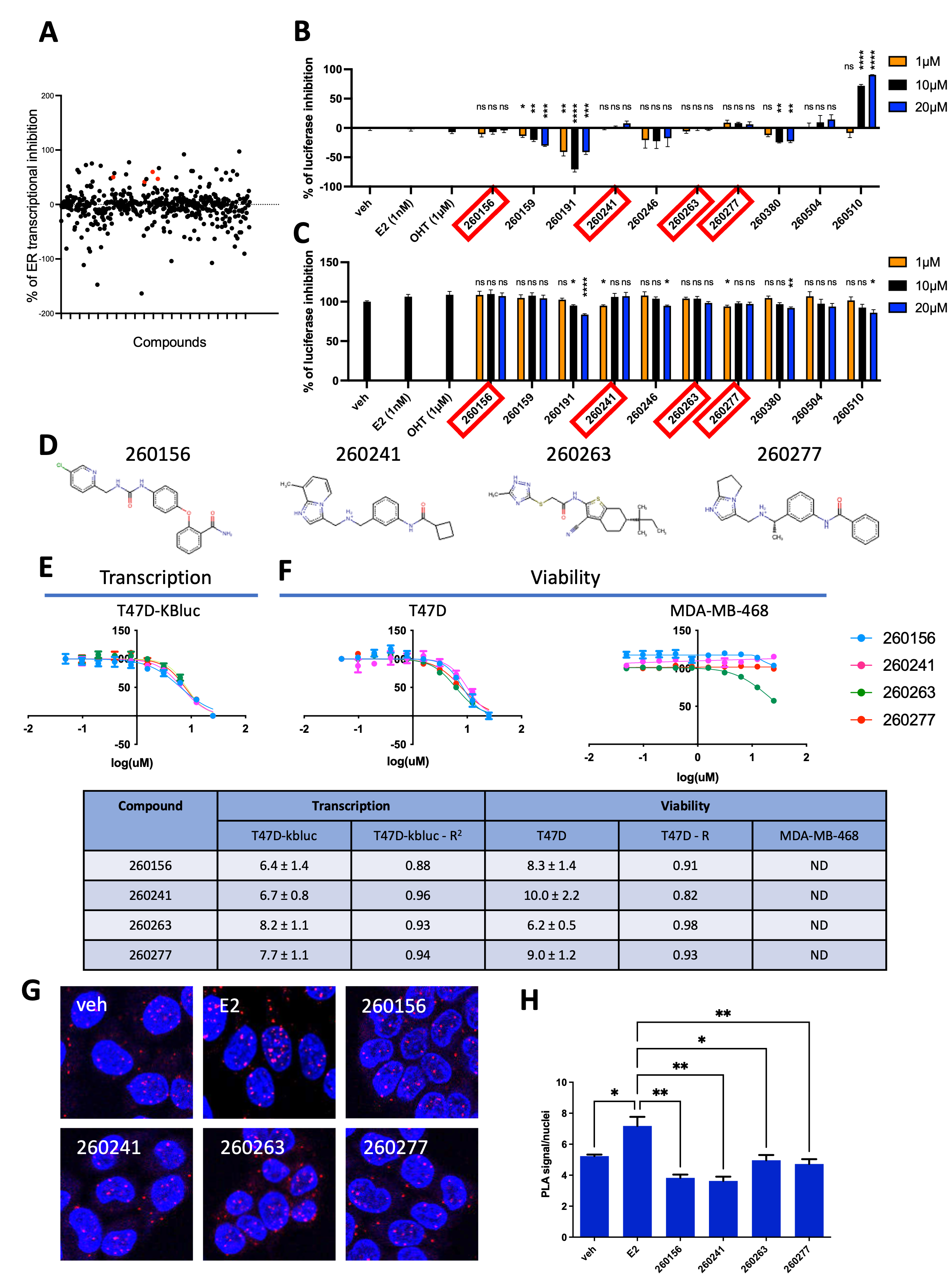

Supplement: Supplementary file 2 — Supplementary Material 2: Supplementary Figure S2. Cell-based data used for in silico similarity searches. (A) The percentage of ER transcriptional inhibition after treatment of T47D-kbluc cells with 10 µM of each of the 512 compounds, identified through in silico docking. (B) Counter screen to determine the effect of the compounds on inhibition of luciferase in ER-negative PC3m-luc cells. (C) Viability of current hits in ER-negative PC3m-luc cells to eliminate toxic compounds. (D) Structures of the four selected chemotypes, VPC-260156, VPC-260241, VPC-260263, VPC-260277, highlighted in red in panels A-C. (E) Dose-response inhibition of transcriptional activity measured with luciferase reporter assay in T47D-KBluc cells following treatment of tested compounds for 24 h. (F) Dose-response inhibition of cell viability measured PrestoBlue in T47D and ER-negative MDA-MB-468 cells following treatment of tested compounds for 72 h. (G) PLA showing interaction of ER and SRC3 in T47D cells with quantification of the corresponding PLA signal/nuclei (H). P values are indicated by stars: ns ≥ 0.05, * 0.01 to 0.05, ** 0.001 to 0.01, *** 0.0001 to 0.001, **** <0.0001. [file 13058_2024_1926_MOESM2_ESM.png]

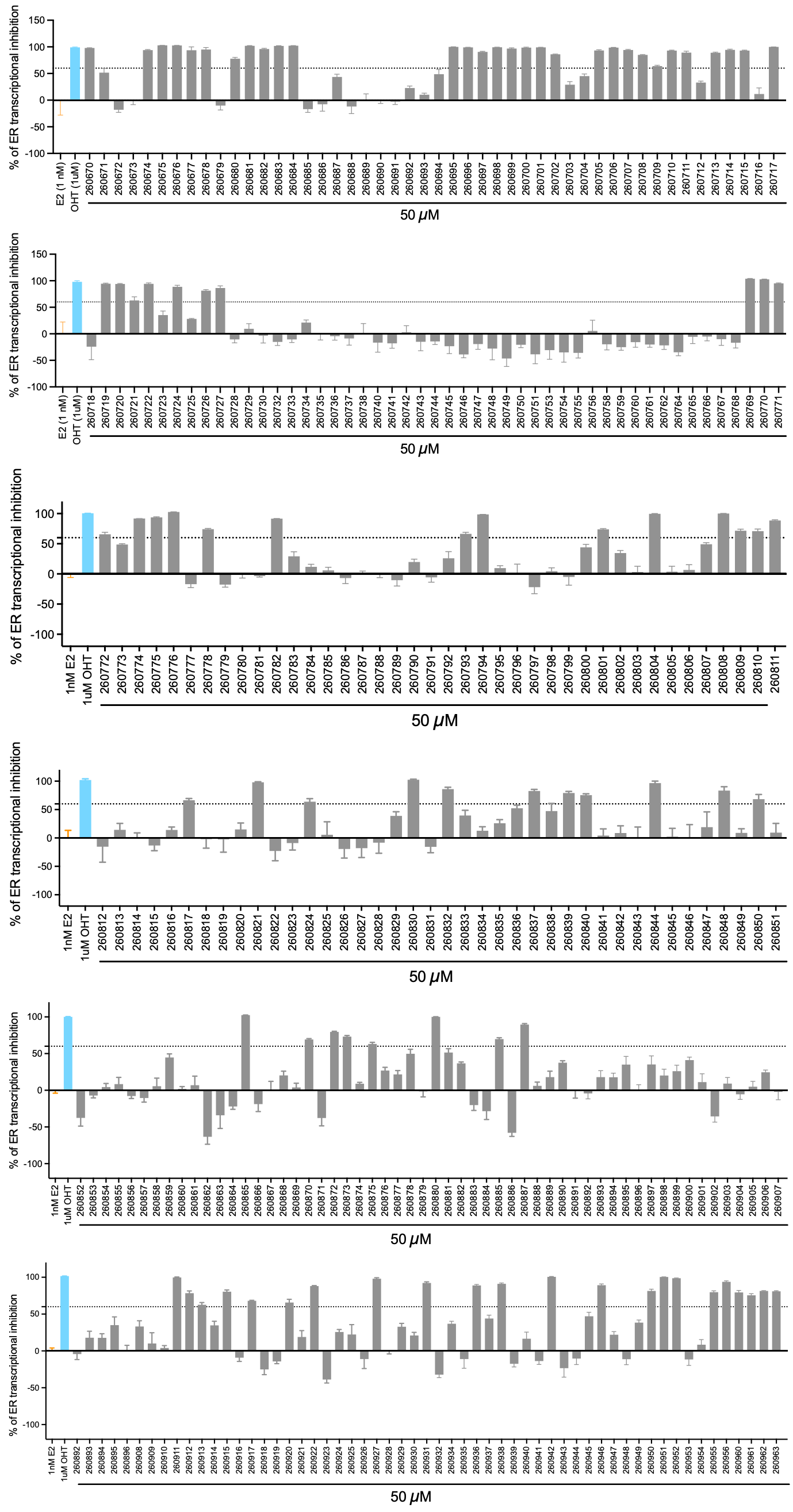

Supplement: Supplementary file 3 — Supplementary Material 3: Supplementary Figure S3. Screening ER inhibitors using a Luciferase reporter-based transcriptional assay. T47D-KBluc cells were treated with 50 µM inhibitor in the presence of 1 nM E2. Luminescence was read after 24 h. Compounds exhibiting >60% inhibition of ERα transcriptional activity were retained as positives for further evaluation. [file 13058_2024_1926_MOESM3_ESM.png]

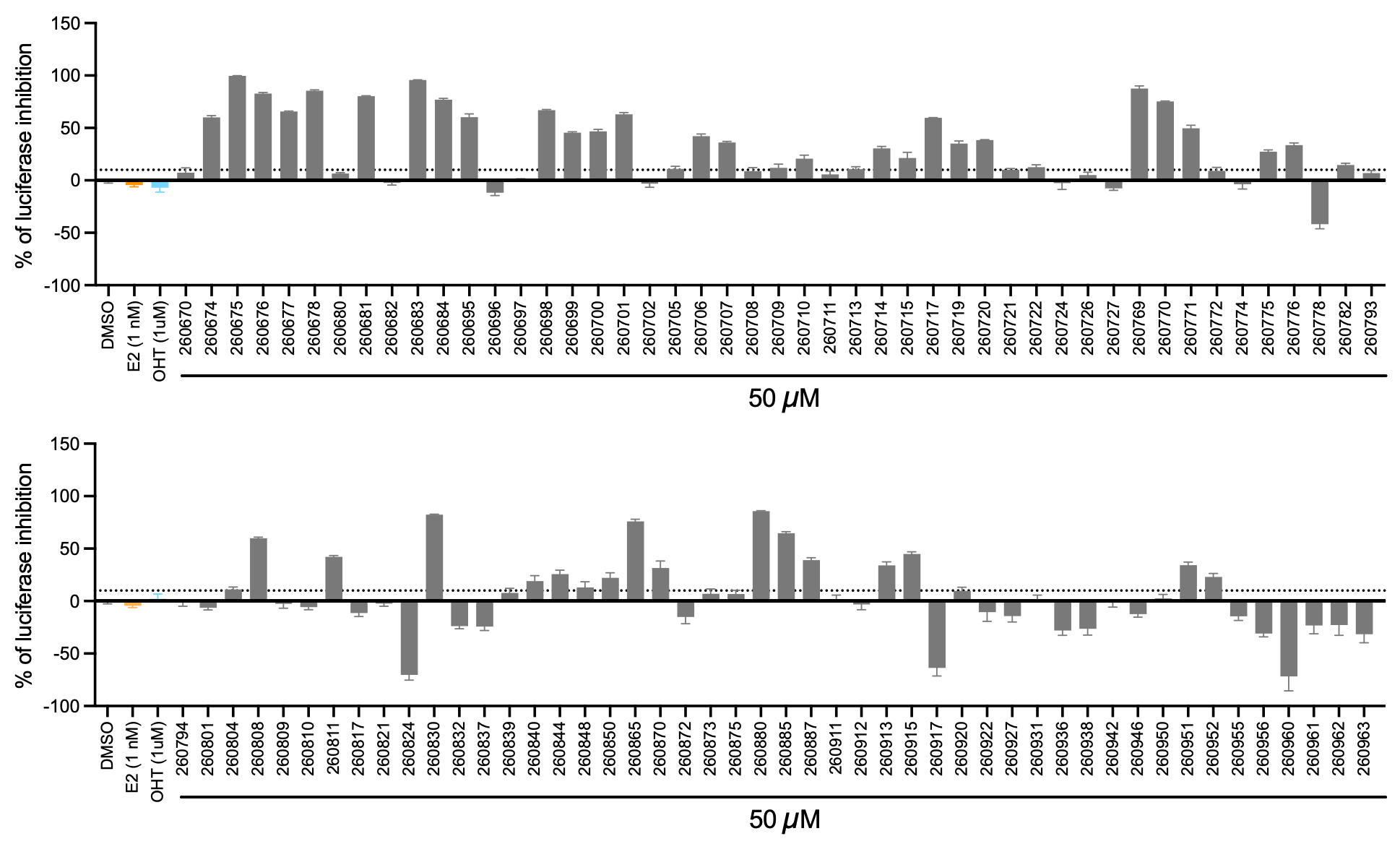

Supplement: Supplementary file 4 — Supplementary Material 4: Supplementary Figure S4. ER-independent inhibition of luciferase enzyme in PC3m-luc cells. To determine if the compounds affected the luciferase enzyme itself or if there were any off-target effects, PC3m-luc cells were treated with 50 µM of tested compounds and luminescence was read after 24 h. Compounds that inhibited luciferase in these cells are acting through a different pathway from ER. Those that exhibited <10% off-target effect was selected for the next screening assay. 38 compounds passed the counter screen. [file 13058_2024_1926_MOESM4_ESM.png]

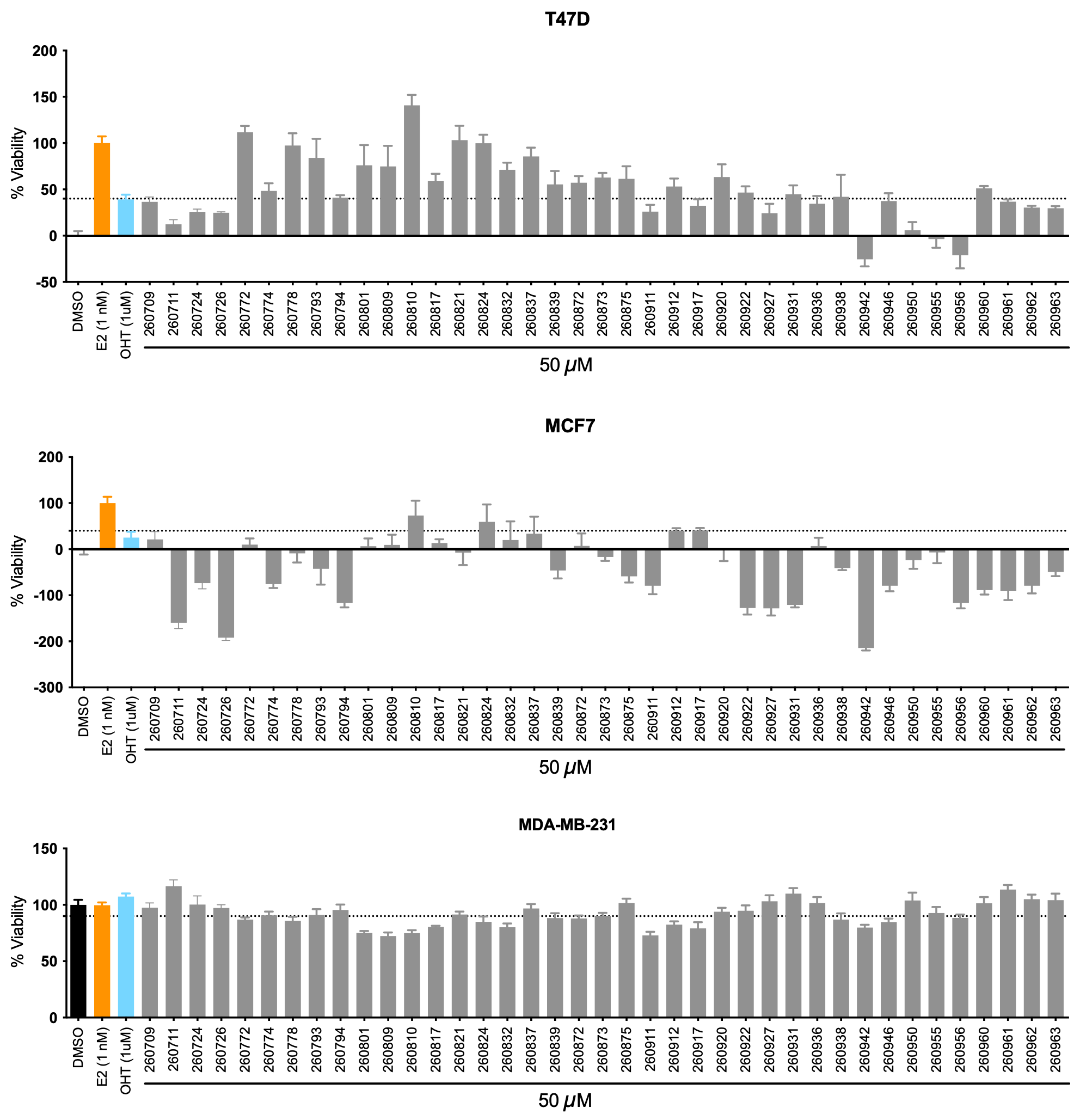

Supplement: Supplementary file 5 — Supplementary Material 5: Supplementary Figure S5. Effect of ER inhibitors on the viability of BCa models. T47D, MCF7, and MDA-MB-231 cells were starved for 4 days and treated with 1 nM E2 and 50 µM of compounds for 72 h. PrestoBlue viability assay determined effect of compounds on viability. Compounds that exhibit inhibitory effect on growth of the ER-positive T47D and MCF7 cells (>60%) and minimal effect on the ER-negative MDA-MB-231 cells (<10%) passed this screen. 11 compounds met this criteria. [file 13058_2024_1926_MOESM5_ESM.png]

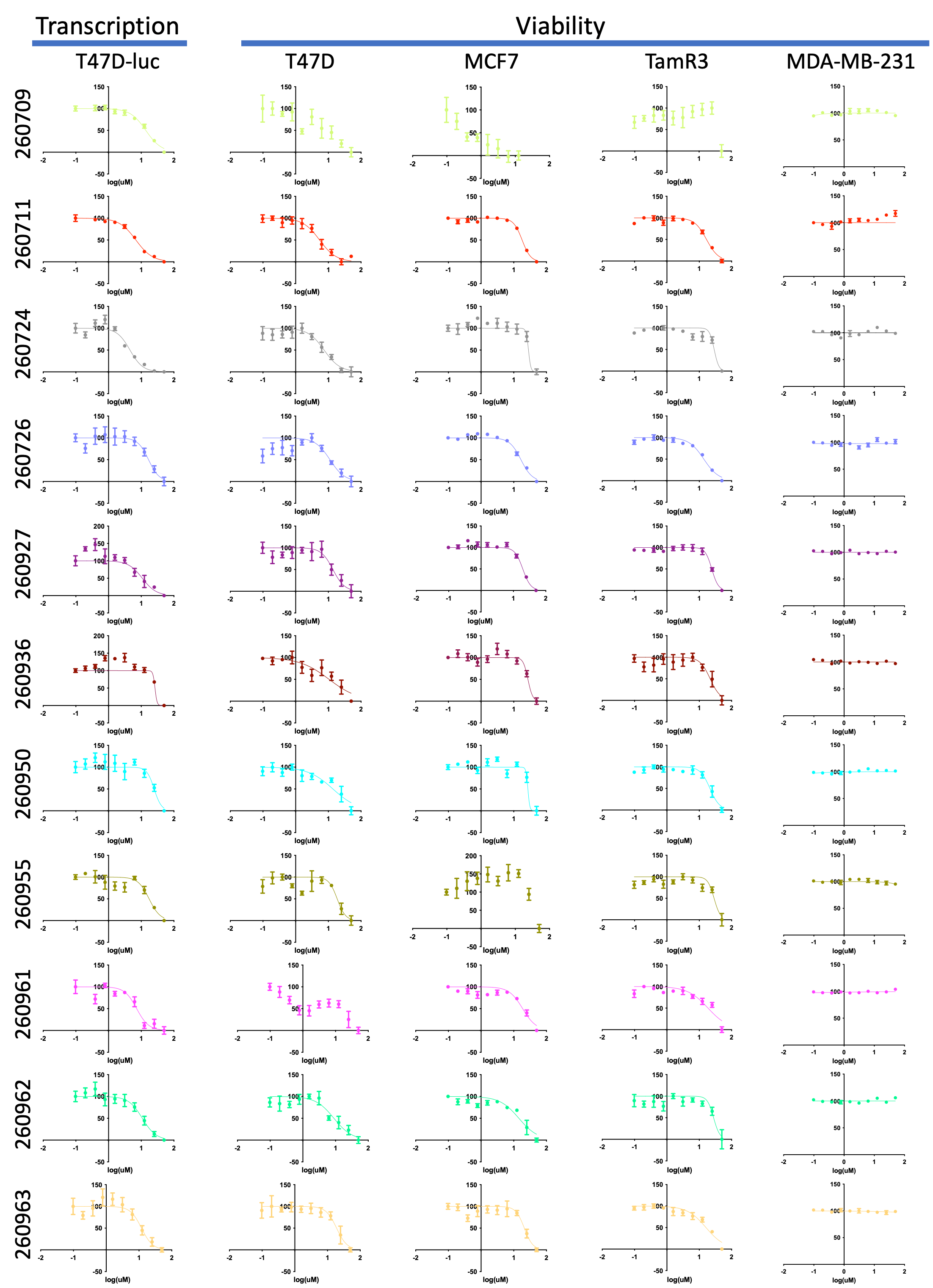

Supplement: Supplementary file 6 — Supplementary Material 6: Supplementary Figure S6. Evaluation of dose response inhibition of ER transcriptional activity and BCa cell viability by lead AF2 compounds. IC50s of lead AF2 compounds in T47D-KBluc, T47D, MCF7, TamR3, and MDA-MB-231 cells were measured by luciferase reporter-based assay for transcription and PrestoBlue assay for viability. Cells were starved for 4 days following 24 h and 72 h treatment for transcriptional and viability inhibition, respectively. [file 13058_2024_1926_MOESM6_ESM.png]

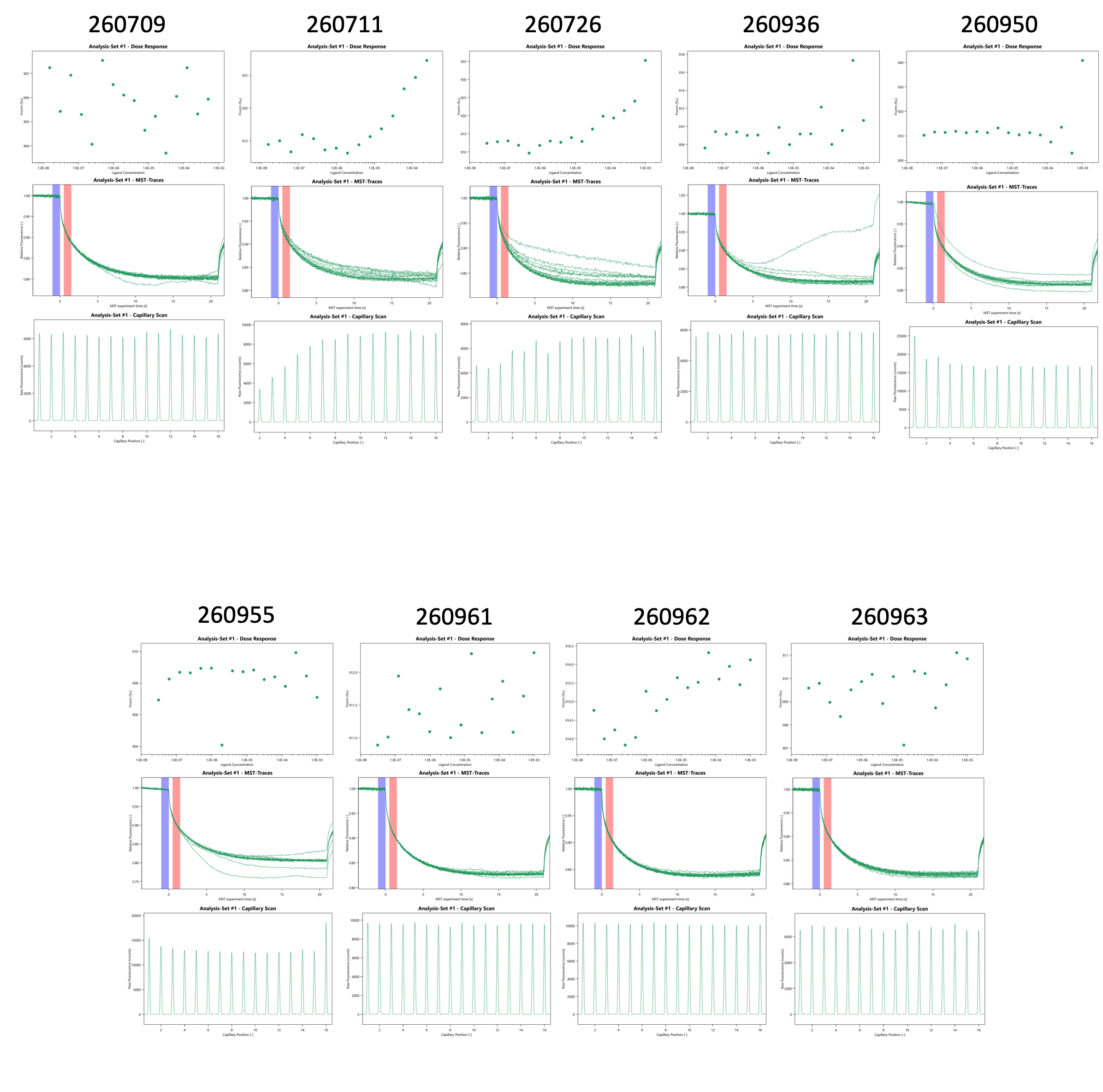

Supplement: Supplementary file 7 — Supplementary Material 7: Supplementary Figure S7. Microscale thermophoresis curves for inactive compounds. Dose-response curves for direct binding of tested compounds with purified ER-LBD measured by MST. 1:2 serially diluted concentrations of lead compounds starting at 1 mM were tested on the movement of fluorescently labeled ER-LBD in a temperature gradient. Compounds shown here did not exhibit direct binding. [file 13058_2024_1926_MOESM7_ESM.png]

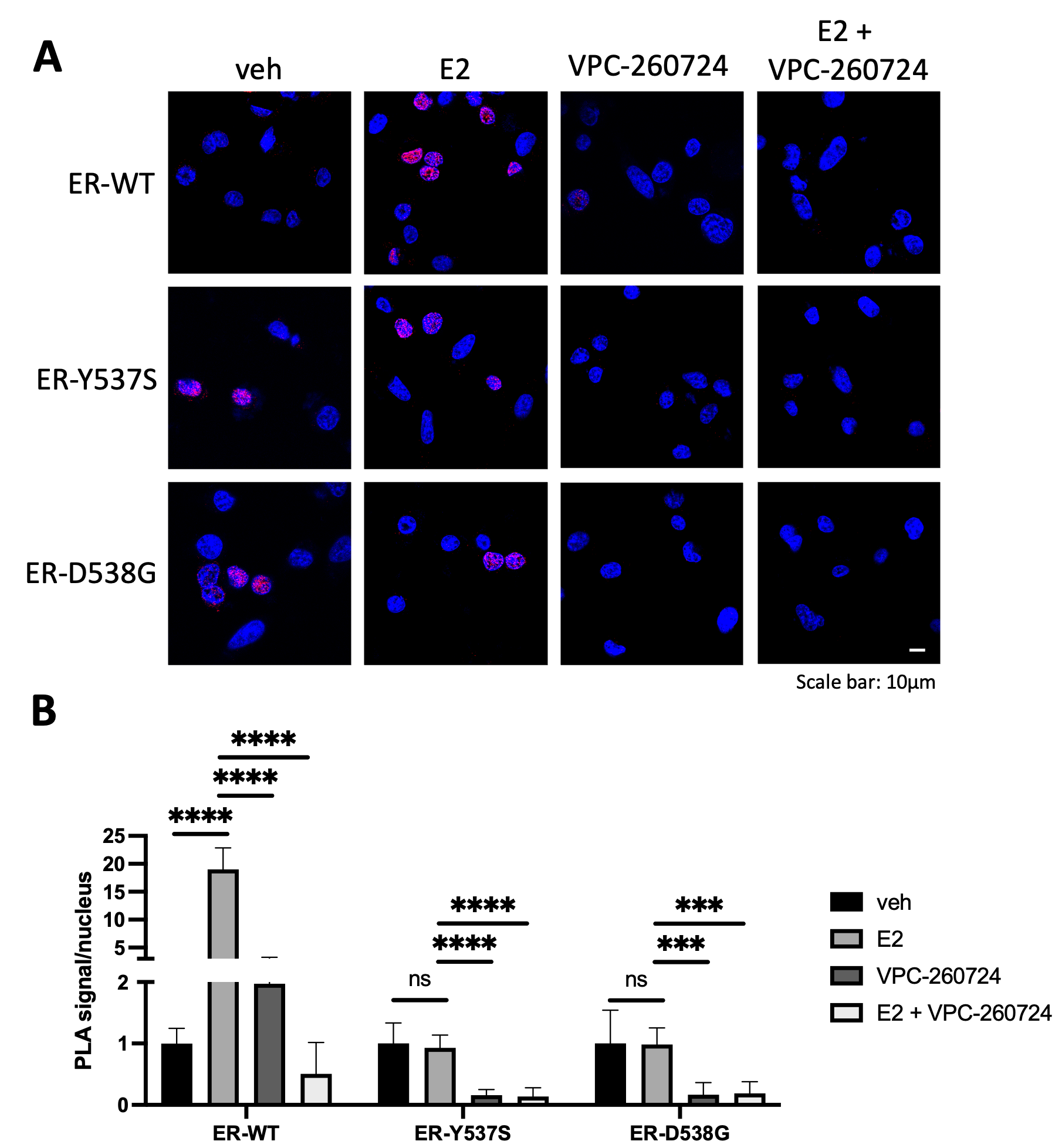

Supplement: Supplementary file 8 — Supplementary Material 8: Supplementary Figure S8. Effect of VPC-260724 on clinically relevant ER mutants. (A) Proximity ligation assay with either ER-WT or ER mutants, Y537S and D538G, and SRC3 treated with 10 µM VPC-260724 in the presence or absence of 1 nM E2. (B) Quantification of ER-WT/mutant – SRC3 interactions as PLA signal/nucleus normalized to vehicle treatment. P values are indicated by stars: ns ≥ 0.05, * 0.01 to 0.05, ** 0.001 to 0.01, *** 0.0001 to 0.001, **** <0.0001. [file 13058_2024_1926_MOESM8_ESM.png]

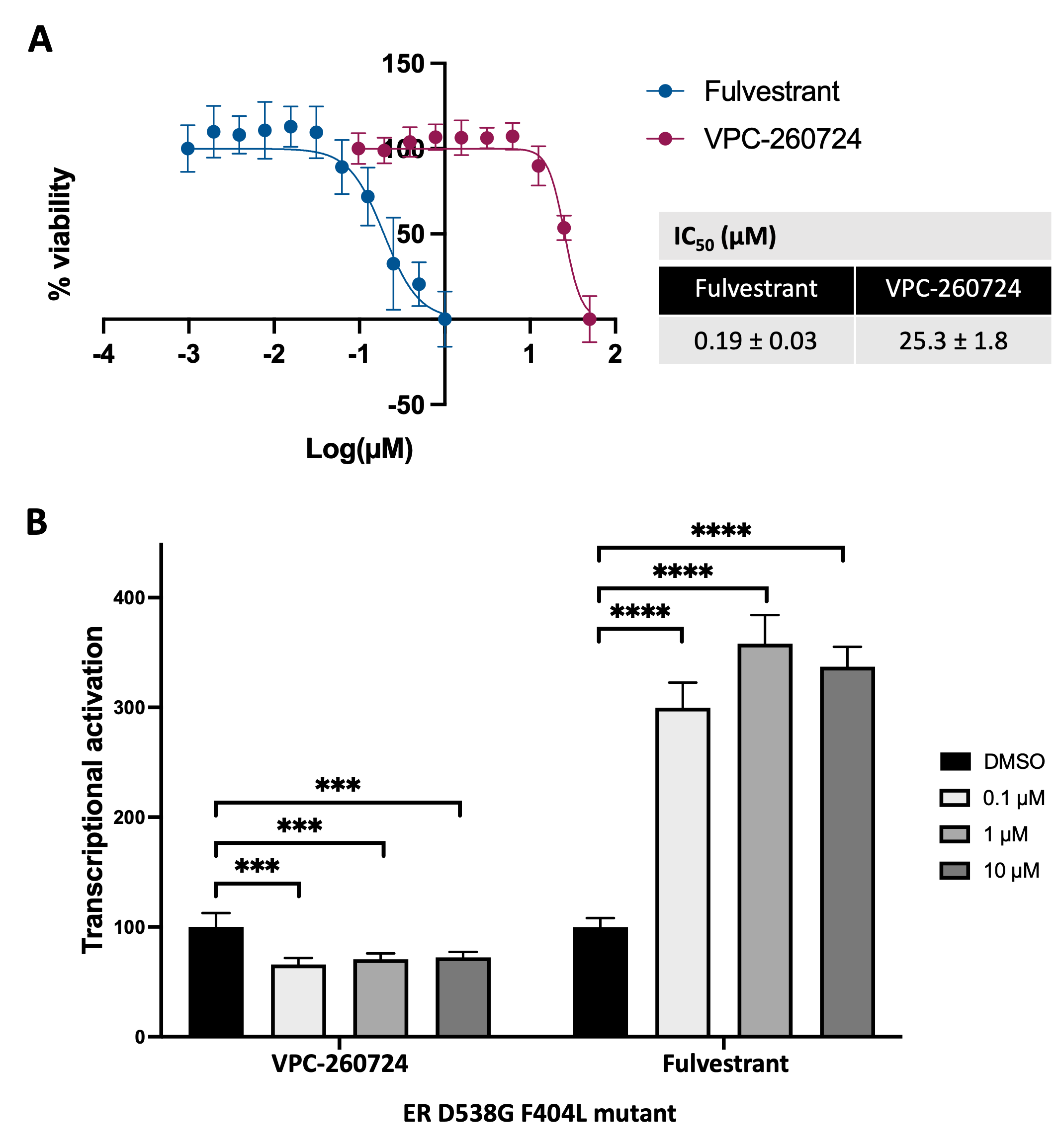

Supplement: Supplementary file 9 — Supplementary Material 9: Supplementary Figure S9. Effect of VPC-260724 on fulvestrant resistant mutant ER D538G/F404L. (A) IC50 of Fulvestrant and VPC-260724 in TamR3 cells were measured by luciferase reporter-based assay for transcription. (B) Effect of VPC-260724 and fulvestrant on transcriptional activity of ER D538/F404L mutant. P values are indicated by stars: ns ≥ 0.05, * 0.01 to 0.05, ** 0.001 to 0.01, *** 0.0001 to 0.001, **** <0.0001. [file 13058_2024_1926_MOESM9_ESM.png]
